# Supplementary material for: Characterizing hypoxia-orchestrated post-stroke changes in oligodendrocyte precursor cells for optimized cell therapy
Source: Stem Cell Reports. 2025 Oct 30;20(11):102687. doi: 10.1016/j.stemcr.2025.102687 (PMC12790735; doi:10.1016/j.stemcr.2025.102687)
Supplement: Document S1. Figures S1–S5, Table S1, and supplemental methods [file mmc1.pdf]

Stem Cell Reports, Volume *20*

Supplemental Information

Characterizing hypoxia-orchestrated post-stroke changes in oligodendrocyte precursor cells for optimized cell therapy

Yasuhiro Kuwata, Ken Yasuda, Kazuto Tsukita, Akihiro Kikuya, Naoki Takayama, Narufumi Yanagida, Kimitoshi Kimura, Ryosuke Takahashi, Riki Matsumoto, and Takakuni Maki

Supplementary Methods

Quality control of single cell RNA sequencing (scRNAseq) data

We conducted a stringent and uniform quality control process for our data according to the single-cell best practices (Heumos et al., 2023), following these steps:

1. Filtering empty droplets: We used the "filtered_bc_matrix" from the Cell Ranger output.
2. Low-quality cell filtering: Outliers were excluded based on the number of detected genes, UMI counts, and mitochondrial percentage, considering values outside the median ± 5 median absolute deviations (MAD).
3. Ambient RNA correction: We used the "raw_bc_matrix" from the Cell Ranger output and corrected for ambient RNA using the SoupX package (version 1.6.2) (Young and Behjati, 2020).
4. Preliminary doublet removal: We removed potential doublets using the scDblFinder package (version 1.14.0) (Germain et al., 2021).

After quality control, we performed count normalization using the "NormalizeData" function in the Seurat package (version 5.0.1), specifying the LogNormalize method with a scale factor of 10,000. We then selected 3,000 variable genes for integration using the "SelectIntegrationFeatures" function in Seurat (version 5.0.1) and integrated all datasets using Harmony (version 1.2.0) (Korsunsky et al., 2019). We applied Uniform Manifold Approximation and Projection (UMAP) dimensionality reduction using Harmony-corrected axes, removed remaining droplets, and annotated cell types based on specific cell markers. Additionally, based on the final annotation, we excluded any datasets containing fewer than 10 Oligodendrocyte Precursor Cells (OPCs). Finally, we conducted unsupervised clustering using the "FindClusters" function (resolution: 1) in the Seurat package (version 5.0.1).

Analyses of transient middle cerebral artery occlusion (tMCAO) atlas

We assessed the function of individual cells by scoring their expression of gene signatures associated with specific biological functions. Gene signatures were primarily retrieved from MSigDB using the msigdbr R package (version 7.5.1) (Castanza et al., 2023) and from the CollecTRI database, which provides comprehensive information on transcription factor regulons (Müller-Dott et al., 2023). To functionally characterize OPCs, we used OPC migration/myelination-associated gene sets (Hamanaka et al., 2023) and OPC-specific HIF-1α-target gene sets (Allan et al., 2021). We also reanalyzed microarray datasets from Olig2cre-DA-cat mice to extract the top 10 differentially expressed genes (DEGs) defining WNT-activated OPCs (Fancy et al., 2009, 2014; Tsai et al., 2016). For defining *ex vivo* severe-hypo-OPCs and mild-hypo-OPCs, we extracted the top 10 DEGs and genes with an adjusted P value < 0.05, respectively. Gene-set scores were calculated using the "AddModuleScore_UCell" function in the UCell R package (version 2.4.0) (Andreatta and Carmona, 2021). For heatmaps, median UCell scores were calculated, normalized, and visualized with color scaling to highlight differences between groups. Statistical analyses were performed using Wilcoxon rank-sum tests, with multiple testing corrections applied using the Benjamini-Hochberg method. For DEG identification, we used the "FindMarkers" function in the Seurat package (version 5.0.1), specifying test.use = "MAST" and min.pct = 0.1 (Finak et al., 2015). To infer cell-cell interactions, we applied the CellChat R package (version 1.6.1) according to its official tutorial (Jin et al., 2023). For trajectory inference, we first conducted a diffusion map using the "scanpy.tl.diffmap" function in Scanpy (version 1.9.6) in Python 3 (version 3.10.13) (Wolf et al.,

2018), followed by trajectory inference and diffusion map pseudotime calculation using the Slingshot R package (version 2.8.0) (Street et al., 2018).

RNA-seq library preparation and sequencing

cDNA libraries were prepared using the Agilent SureSelect Strand Specific RNA Prep Kit (Catalog no. G9691A) with 200 ng of total RNA, following the manufacturer's guidelines. Sequencing was performed on an Illumina MiSeq platform, generating 76 bp paired-end reads with multiplexing.

Processing of bulk RNAseq data

We also applied uniform and stringent data processing to all bulk RNA-seq datasets. Adapter sequences were removed, and reads were quality-trimmed using Trimmomatic (version 0.36). Post-trimming, read quality was assessed with FastQC (version 0.11.9). The primary assembly version of the Rattus norvegicus reference genome (mRatBN7.2) was obtained from the Ensembl database. Trimmed reads were then aligned to the reference genome using STAR aligner (version 2.7.9a), and gene expression levels were quantified using RSEM (version 1.3.3).

Analyses of bulk RNAseq data

For differential gene expression analysis, we used the edgeR package (version 3.42.4). Gene counts were normalized using the "calcNormFactors" function, and low-expression genes were filtered by retaining only those with a counts-per-million value above 10 in at least one sample. A linear model was fitted using the voom method from the limma package (version 3.56.2), and differential expression was tested using an empirical Bayes approach. For gene set enrichment

analysis (GSEA), gene signatures were extracted as detailed in the "Analyses of tMCAO Atlas" subsection and analyzed using the "GSEA" function from the clusterProfiler R package (version 4.8.3).

Other publicly available data analyzed in this study

In addition to the data used to create the tMCAO atlas, we analyzed the following scRNA-seq and bulk RNA-seq datasets:

• GSE114609: A bulk RNA-seq dataset of ex vivo severe-hypo-OPCs previously generated by our group (Kishida et al., 2019).

• GSE19403: A microarray dataset of *in vivo* OPCs from WNT-β-catenin constitutively activated mice (Fancy et al., 2009, 2014).

• GSE75330: An scRNA-seq dataset of *in vivo* OPCs from spinal cords (Marques et al., 2016).

• GSE173182: A bulk RNA-seq dataset of *ex vivo* OPCs treated with IFN-γ or dexamethasone (Meijer et al., 2022).

• Multi-organ endothelial cell atlas: An scRNA-seq dataset of endothelial cells from seven organs (Bondareva et al., 2022).

For these datasets, we downloaded the corresponding FASTQ files and processed them through the pipeline described earlier.

Supplementary References

Allan, K.C., Hu, L.R., Scavuzzo, M.A., Morton, A.R., Gevorgyan, A.S., Cohn, E.F., Clayton, B.L.L., Bederman, I.R., Hung, S., Bartels, C.F., et al. (2021). Non-canonical targets of HIF1a impair oligodendrocyte progenitor cell function. Cell Stem Cell 28, 257–272.e11.

Andreatta, M., and Carmona, S.J. (2021). UCell: robust and scalable single-cell gene signature scoring. Comput. Struct. Biotechnol. J. 19, 3796–3798.

Bondareva, O., Rodríguez-Aguilera, J.R., Oliveira, F., Liao, L., Rose, A., Gupta, A., Singh, K., Geier, F., Schuster, J., Boeckel, J.-N., et al. (2022). Single-cell profiling of vascular endothelial cells reveals progressive organ-specific vulnerabilities during obesity. Nat. Metab. 4, 1591–1610.

Castanza, A.S., Recla, J.M., Eby, D., Thorvaldsdóttir, H., Bult, C.J., and Mesirov, J.P. (2023). Extending support for mouse data in the Molecular Signatures Database (MSigDB). Nat. Methods 20, 1619–1620.

Fancy, S.P.J., Baranzini, S.E., Zhao, C., Yuk, D.-I., Irvine, K.-A., Kaing, S., Sanai, N., Franklin, R.J.M., and Rowitch, D.H. (2009). Dysregulation of the Wnt pathway inhibits timely myelination and remyelination in the mammalian CNS. Genes Dev. 23, 1571–1585.

Fancy, S.P.J., Harrington, E.P., Baranzini, S.E., Silbereis, J.C., Shiow, L.R., Yuen, T.J., Huang, E.J., Lomvardas, S., and Rowitch, D.H. (2014). Parallel states of pathological Wnt signaling in neonatal brain injury and colon cancer. Nat. Neurosci. 17, 506–512.

Finak, G., McDavid, A., Yajima, M., Deng, J., Gersuk, V., Shalek, A.K., Slichter, C.K., Miller, H.W., McElrath, M.J., Prlic, M., et al. (2015). MAST: a flexible statistical framework for assessing transcriptional changes and characterizing heterogeneity in single-cell RNA sequencing data. Genome Biol. 16, 278.

Germain, P.-L., Lun, A., Garcia Meixide, C., Macnair, W., and Robinson, M.D. (2021). Doublet identification in single-cell sequencing data using scDblFinder. F1000Res 10, 979.

Hamanaka, G., Hernández, I.C., Takase, H., Ishikawa, H., Benboujja, F., Kimura, S., Fukuda, N., Guo, S., Lok, J., Lo, E.H., et al. (2023). Myelination- and migration-associated genes are downregulated after phagocytosis in cultured oligodendrocyte precursor cells. J. Neurochem. 167, 571–581.

Heumos, L., Schaar, A.C., Lance, C., Litinetskaya, A., Drost, F., Zappia, L., Lücken, M.D., Strobl, D.C., Henao, J., Curion, F., et al. (2023). Best practices for single-cell analysis across modalities. Nat. Rev. Genet. 24, 550–572.

Jin, S., Plikus, M.V., and Nie, Q. (2023). CellChat for systematic analysis of cell-cell communication from single-cell and spatially resolved transcriptomics. bioRxiv. https://doi.org/10.1101/2023.11.05.565674

Kishida, N., Maki, T., Takagi, Y., Yasuda, K., Kinoshita, H., Ayaki, T., Noro, T., Kinoshita, Y., Ono, Y., Kataoka, H., et al. (2019). Role of perivascular oligodendrocyte precursor cells in angiogenesis after brain ischemia. J. Am. Heart Assoc. 8, e011824.

Korsunsky, I., Millard, N., Fan, J., Slowikowski, K., Zhang, F., Wei, K., Baglaenko, Y., Brenner, M., Loh, P.-R., and Raychaudhuri, S. (2019). Fast, sensitive and accurate integration of single-cell data with Harmony. Nat. Methods 16, 1289–1296.

Marques, S., Zeisel, A., Codeluppi, S., van Bruggen, D., Mendanha Falcão, A., Xiao, L., Li, H., Häring, M., Hochgerner, H., Romanov, R.A., et al. (2016). Oligodendrocyte heterogeneity in the mouse juvenile and adult central nervous system. Science 352, 1326–1329.

Meijer, M., Agirre, E., Kabbe, M., van Tuijn, C.A., Heskol, A., Zheng, C., Mendanha Falcão, A., Bartosovic, M., Kirby, L., Calini, D., et al. (2022). Epigenomic priming of immune

genes implicates oligodendroglia in multiple sclerosis susceptibility. Neuron 110, 1193–1210.e13.

Müller-Dott, S., Tsirvouli, E., Vazquez, M., Ramirez Flores, R.O., Badia-I-Mompel, P., Fallegger, R., Türei, D., Lægreid, A., and Saez-Rodriguez, J. (2023). Expanding the coverage of regulons from high-confidence prior knowledge for accurate estimation of transcription factor activities. Nucleic Acids Res. 51, 10934–10949.

Street, K., Risso, D., Fletcher, R.B., Das, D., Ngai, J., Yosef, N., Purdom, E., and Dudoit, S. (2018). Slingshot: cell lineage and pseudotime inference for single-cell transcriptomics. BMC Genomics 19, 477.

Tsai, H.-H., Niu, J., Munji, R., Davalos, D., Chang, J., Zhang, H., Tien, A.-C., Kuo, C.J., Chan, J.R., Daneman, R., et al. (2016). Oligodendrocyte precursors migrate along vasculature in the developing nervous system. Science 351, 379–384.

Wolf, F.A., Angerer, P., and Theis, F.J. (2018). SCANPY: large-scale single-cell gene expression data analysis. Genome Biol. 19, 15.

Young, M.D., and Behjati, S. (2020). SoupX removes ambient RNA contamination from droplet-based single-cell RNA sequencing data. Gigascience 9, giaa151.

Table S1. Single-cell RNA sequencing datasets considered in this paper.

BioProject, project ID	Sample ID	Mouse (strain, genotype, age, sex)	Characteristics	FACS	Ischemia duration	Included?
PRJNA597034	GSM4227433	C57BL/6, WT, 6 weeks, male	Contralateral	Not applied	1.0 hour	No[1]
PRJNA597034	GSM4227434	C57BL/6, WT, 6 weeks, male	post-tMCAO 4 hrs	Not applied	1.0 hour	No[2]
PRJNA597034	GSM4227435	C57BL/6, WT, 6 weeks, male	Contralateral	Not applied	1.0 hour	No[1]
PRJNA597034	GSM4227436	C57BL/6, WT, 6 weeks, male	post-tMCAO 1 days	Not applied	1.0 hour	Yes
PRJNA597034	GSM4227437	C57BL/6, WT, 6 weeks, male	Contralateral	Not applied	1.0 hour	No[1]
PRJNA597034	GSM4227438	C57BL/6, WT, 6 weeks, male	post-tMCAO 3 days	Not applied	1.0 hour	Yes
PRJNA597034	GSM4227439	C57BL/6, WT, 6 weeks, male	Contralateral	Not applied	1.0 hour	No[1]
PRJNA597034	GSM4227440	C57BL/6, WT, 6 weeks, male	post-tMCAO 7 days	Not applied	1.0 hour	Yes
PRJNA597034	GSM4227441	C57BL/6, WT, 6 weeks, male	Sham	Not applied	–	Yes
PRJNA597034	GSM4227442	C57BL/6, WT, 6 weeks, male	Sham	Not applied	–	Yes
PRJNA597034	GSM4227443	C57BL/6, Cd300a-fl/f, 6 weeks, male	Sham	Not applied	–	Yes
PRJNA646185	GSM4670418	C57BL/6, Cd300a-fl/f, NS, NS	Sham	CD45-positive	–	No[3]
PRJNA646185	GSM4670420	C57BL/6, Cd300a-fl/f, NS, NS	post-tMCAO 1 hr	CD45-positive	1.0 hour	No[3]
PRJNA646185	GSM4670422	C57BL/6, Cd300a-fl/f, NS, NS	post-tMCAO 3 hrs	CD45-positive	1.0 hour	No[3]
PRJNA705094	GSM5111158	C57BL/6, WT, 10 weeks, male	Sham	Not applied	–	Yes
PRJNA705094	GSM5111159	C57BL/6, WT, 10 weeks, male	post-tMCAO 14 days	Not applied	1.5 hour	Yes
PRJNA718585	GSM5220257	C57BL/6, WT, NS, NS	post-tMCAO 5 days	CD45-positive	1.0 hour	No[3]
PRJNA718585	GSM5220258	C57BL/6, WT, NS, NS	post-tMCAO 5 days	CD45-positive	1.0 hour	No[3]
PRJNA718585	GSM5220259	C57BL/6, WT, NS, NS	post-tMCAO 5 days	CD45-positive	1.0 hour	No[3]
PRJNA718585	GSM5220260	C57BL/6, WT, NS, NS	post-tMCAO 5 days	CD45-positive	1.0 hour	No[3]
PRJNA730565	GSM5319987	C57BL/6, WT, 6-8 weeks, NS	Sham	Not applied	–	Yes
PRJNA730565	GSM5319988	C57BL/6, WT, 6-8 weeks, NS	Sham	Not applied	–	Yes

PRJNA730565	GSM5319989	C57BL/6, WT, 6-8 weeks, NS	Sham	Not applied	–	Yes
PRJNA730565	GSM5319990	C57BL/6, WT, 6-8 weeks, NS	post-tMCAO 1 day	Not applied	1.0 hour	Yes
PRJNA730565	GSM5319991	C57BL/6, WT, 6-8 weeks, NS	post-tMCAO 1 day	Not applied	1.0 hour	Yes
PRJNA730565	GSM5319992	C57BL/6, WT, 6-8 weeks, NS	post-tMCAO 1 day	Not applied	1.0 hour	Yes
PRJNA783004	GSM5701739	C57BL/6, WT, NS, NS	Sham	CD45-positive	–	No[3]
PRJNA783004	GSM5701741	C57BL/6, WT, NS, NS	Sham	CD45-positive	–	No[3]
PRJNA783004	GSM5701742	C57BL/6, WT, NS, NS	post-tMCAO 1 day	CD45-positive	0.5–0.75 hours	No[3]
PRJNA783004	GSM5701746	C57BL/6, WT, NS, NS	post-tMCAO 3 day	CD45-positive	0.5–0.75 hours	No[3]
PRJNA811768	GSM5701743	C57BL/6, WT, NS, NS	post-tMCAO 1 day	Not applied	1.0 hour	No[3]
PRJNA811768	GSM5929214	C57BL/6, WT, NS, NS	post-tMCAO 1 day	Not applied	1.0 hour	No[3]
PRJNA811768	GSM5929217	C57BL/6, WT, NS, NS	Sham	Not applied	–	No[3]
PRJNA811768	GSM5929218	C57BL/6, WT, NS, NS	post-tMCAO 2 days	Not applied	1.0 hour	No[3]
PRJNA858806	GSM6339290	C57BL/6, WT, NS, NS	Contralateral	A2B5, O4, CD45-positive cells	1.0 hour	No[1]
PRJNA858806	GSM6339291	C57BL/6, WT, NS, NS	post-tMCAO 14 days	A2B5, O4, CD45-positive cells	1.0 hour	Yes
PRJNA946281	GSM7104632	C57BL/6, WT, 8 weeks, NS	post-tMCAO 1 day	Not applied	1.5 hour	Yes
PRJNA946281	GSM7104633	C57BL/6, WT, 8 weeks, NS	post-tMCAO 3 days	Not applied	1.5 hour	Yes
PRJNA946281	GSM7104634	C57BL/6, WT, 8 weeks, NS	post-tMCAO 7 days	Not applied	1.5 hour	Yes
PRJNA946281	GSM7104635	C57BL/6, WT, 8 weeks, NS	Sham	Not applied	–	Yes
PRJEB42257	SAMEA7747040	C57BL/6, WT, NS, male	Sham	CD31/45-negative	–	Yes
PRJEB42257	SAMEA7747041	C57BL/6, WT, NS, male	post-tMCAO 3 days	CD31/45-negative	NS	Yes

[1]Contralateral hemisphere, [2]No other datasets collected in this time point, [3]Not sufficient OPCs.
Abbreviations: FACS, Fluorescence-Activated Cell Sorting; WT, Wild Type; NS, Not Specified; tMCAO, transient Middle Cerebral Artery Occlusion.

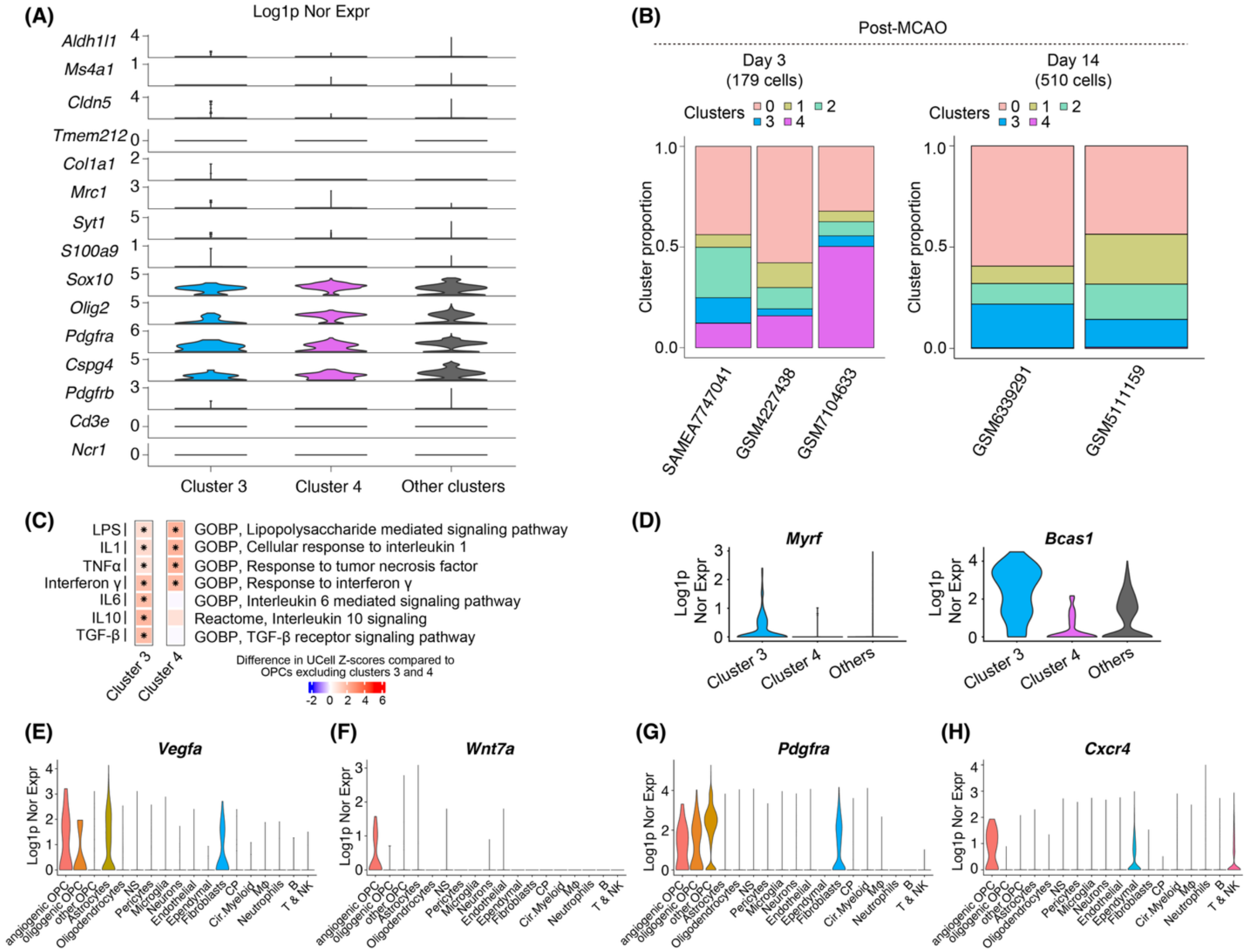

Fig. S1. Characterization of clusters 3 and 4 within oligodendrocyte precursor cells (OPCs). (A) OPC subclusters, including clusters 3 and 4, do not express genes specific to non-OPC cell types. (B) An increase in the proportion of clusters 3 and 4 is observed across all samples, with cluster 4 expanding at 3 days post-transient middle cerebral artery occlusion (tMCAO) and cluster 3 at 14 days post-tMCAO. Abbreviations: Log1p Nor Expr, Log1p-transformed normalized expression. (C) Both clusters are significantly influenced by various cytokines. Notably, cluster 4, corresponding to “angiogenic” OPCs, is more affected by cytokines involved in the acute inflammatory response, such as interleukin (IL)-1β and tumor necrosis factor (TNF)-α. In contrast, cluster 3, corresponding to “oligogenic” OPCs, is specifically influenced by IL-

10 and TGF-β. (D) *Myrf* and *Bcas1*, genes reported to be crucial for OPC maturation, are specifically expressed in cluster 3, with *Myrf* showing cluster-specific expression and Bcas1 being upregulated. (E, F) Cluster 4 of OPCs, corresponding to "angiogenic" OPCs, upregulates *Vegfa* (E) and *Wnt7a* (F), even compared to all other cell types. (G, H) *Pdgfra*, a marker gene for OPCs (G), and *Cxcr4*, a marker gene distinguishing "angiogenic" OPCs from other OPC types (H), distinctively mark "angiogenic" OPCs. Abbreviations: Log1p Nor Expr, Log1p-transformed normalized expression; GOBP, Gene Ontology Biological Process; Reactome, Reactome pathway database; NS, Neural Stem cells; CP, Choroid Plexus cells; Cir.Myeloid, Circulating Myeloid cells; Mφ, Macrophages; B, B cells; T & NK, T cells and NK cells.

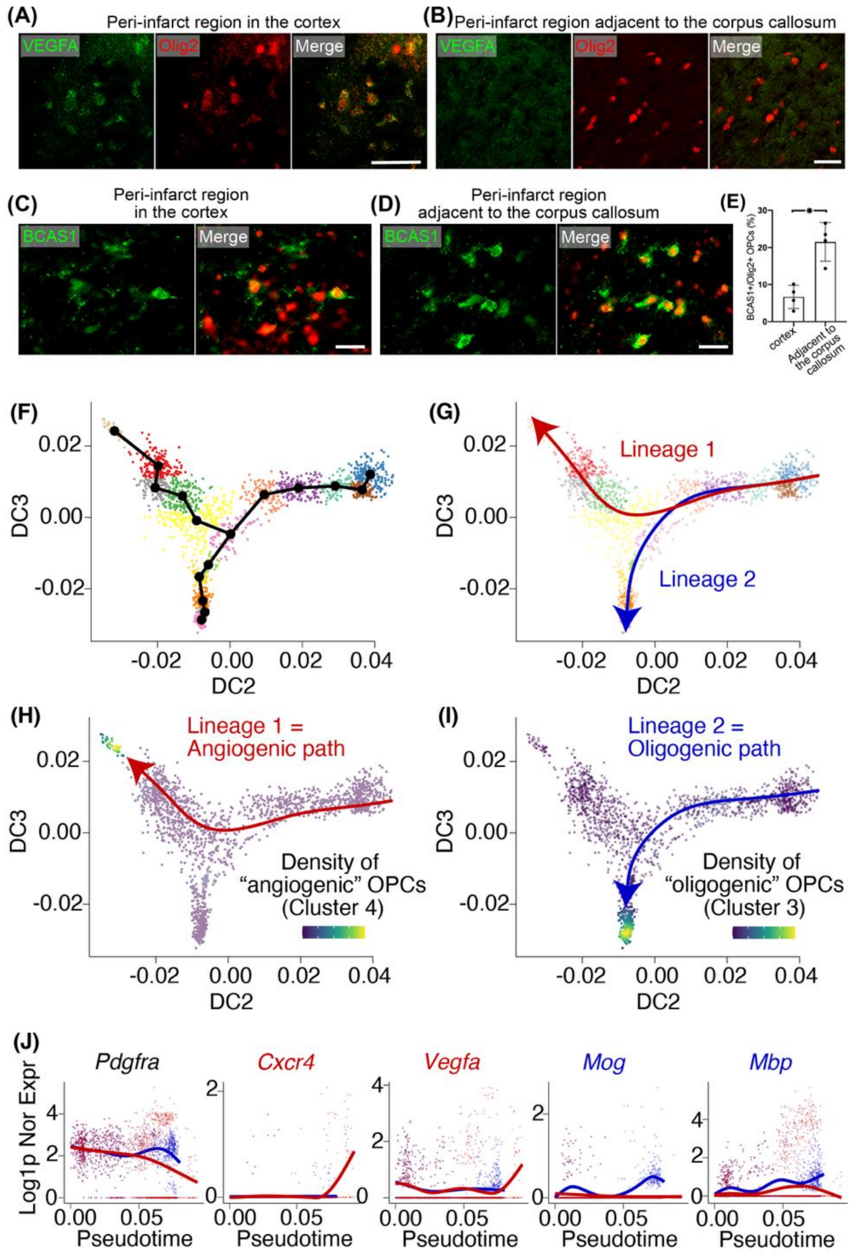

Fig. S2. Spatial distribution and trajectory analysis of angiogenic and oligogenic oligodendrocyte precursor cells (OPCs) following transient middle cerebral artery occlusion (tMCAO). (A, B) Representative immunofluorescence images showing VEGFA^{+} OLIG2^{+} "angiogenic" OPCs at day 3 post-tMCAO in the peri-infarct cortex (A) and the peri-infarct corpus callosum with its adjacent regions (B). (C, D) Representative immunofluorescence

images showing BCAS1$^+$ OLIG2$^+$ "oligogenic" OPCs at day 14 post-MCAO in the peri-infarct cortex (C) and the peri-infarct corpus callosum with its adjacent regions (D). (E) Quantification of the proportions of BCAS1$^+$ OLIG2$^+$ "oligogenic" OPCs (day 14) among total OLIG2$^+$ oligodendrocyte lineage cells in the peri-infarct cortex versus peri-infarct corpus callosum with its adjacent regions. N = 4. Scale bars = 50 µm. (F) Dimensional reduction using diffusion maps, followed by trajectory inference, identified a single branching point. (G) The "getCurves" function in the Slingshot R package revealed two distinct paths. (H, I) One path terminates in angiogenic OPCs, while the other terminates in oligogenic OPCs. (J) Gene expression changes along the diffusion map pseudotime show an increase in *Cxcr4* and *Vegfa* along the angiogenic path, whereas *Mog* and *Mbp* increase along the oligogenic path. Abbreviations: DC, Diffusion Component; Log1p Nor Expr, Log1p-transformed normalized expression.

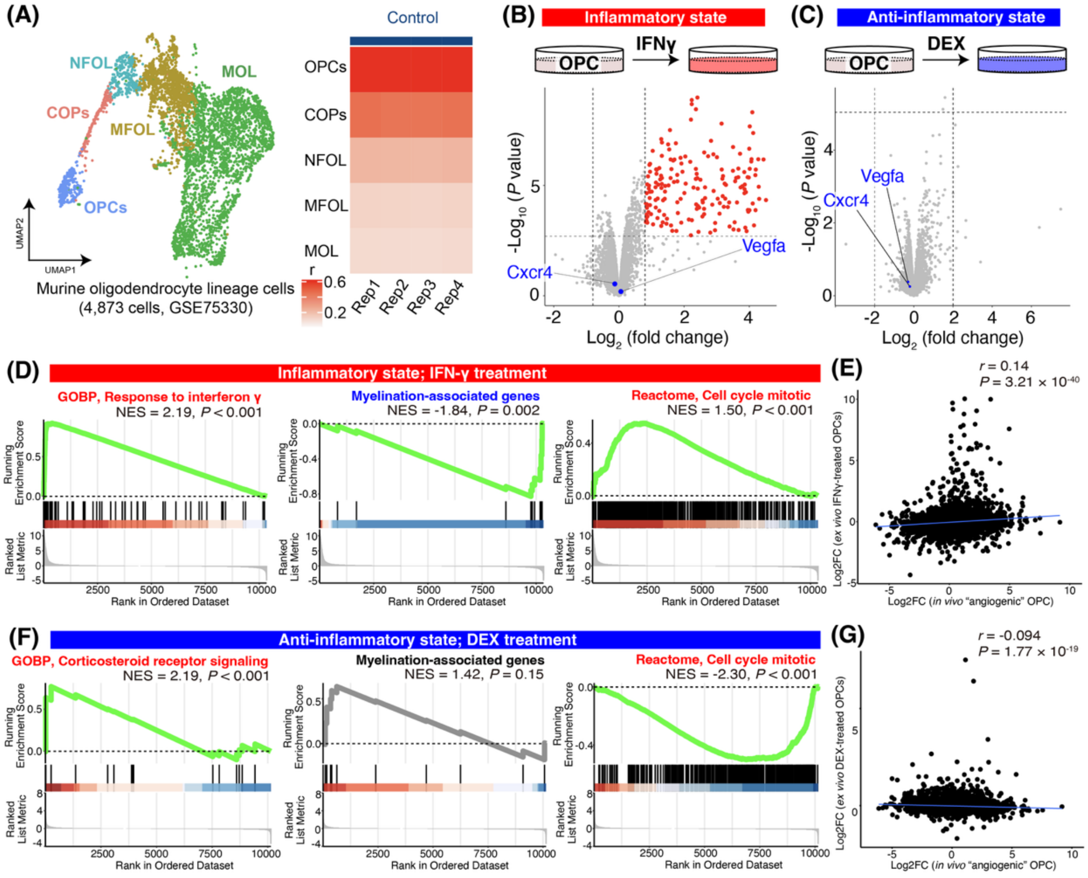

Fig. S3. Validation of *ex vivo* oligodendrocyte precursor cells (OPC) cultures and their transcriptomic responses to inflammatory and anti-inflammatory states. (A) Correlation analysis revealed that our *ex vivo* OPC cultures accurately capture the transcriptional profile of *in vivo* OPCs in the murine brain. (B, C) Inflammatory and anti-inflammatory states were induced using interferon-γ (IFN-γ) and dexamethasone (DEX), respectively. Differentially expressed gene (DEG) analysis revealed no significant transcriptomic changes in *Vegfa* and *Cxcr4*. (D) The inflammatory state led to the downregulation of myelination-associated genes and the upregulation of mitosis-associated genes. (E) Correlation analysis revealed weak but significant positive correlations of log2 fold changes (log2FC) between *in vivo* "angiogenic" OPCs and *ex vivo* IFN-γ-treated OPCs. (F) The anti-inflammatory state resulted in the downregulation of mitosis-associated genes. (G) Correlation analysis revealed weak but significant negative

correlations of log2FC between *in vivo* “angiogenic” OPCs and *ex vivo* DEX-treated OPCs. Abbreviations: GSE, Gene expression omnibus SEries; COPs, Committed Oligodendrocyte Precursors; NFOL, Newly Forming Oligodendrocytes; MFOL, Myelin-Forming Oligodendrocytes; MOL, Mature Oligodendrocytes; GOBP, Gene Ontology Biological Process; Reactome, Reactome pathway database; NES, Normalized Enrichment Score; *rho*, Spearman’s rank correlation coefficient.

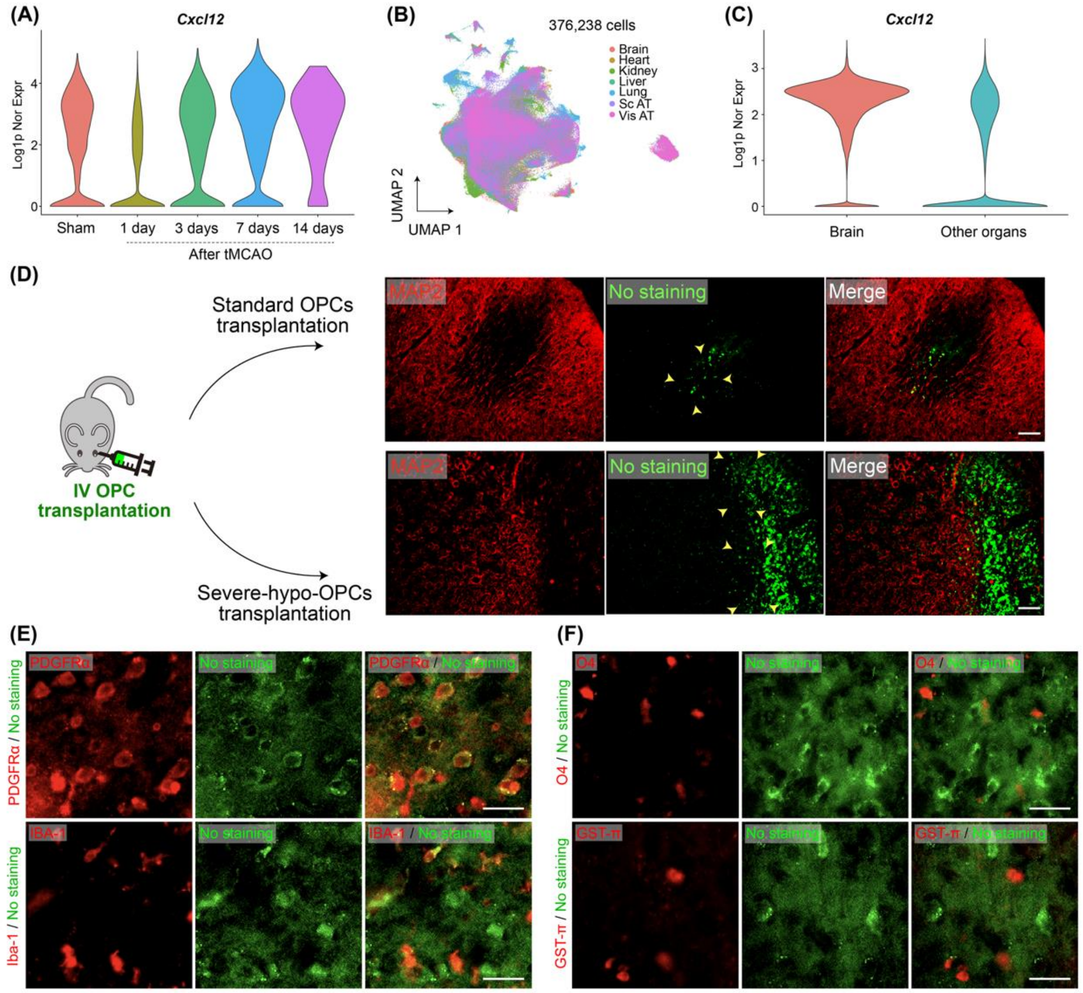

Fig. S4. Brain endothelial *Cxcl12* expression and behavior of transplanted oligodendrocyte precursor cells (OPCs) in the ischemic brain. (A) *Cxcl12* expression in endothelial cells was upregulated 3 days post-tMCAO. (B, C) Reanalysis of a publicly available cross-organ endothelial cell atlas (B) revealed that brain endothelial cells exhibit high *Cxcl12* expression (C). (D) Representative immunofluorescence images of EGFP-labeled standard OPCs and OPCs subjected to severe hypoxic preconditioning *ex vivo* (severe-hypo-OPCs), shown at day 14 after intravenous transplantation (3 days post-MCAO). GFP-positive transplanted OPCs were detected without additional anti-GFP staining ("No staining"). GFP^{+} transplanted cells were observed in the ischemic border and peri-infarct penumbra (regions outlined by arrowheads). Please note that

the lesion shown in the upper panel represents a small remote infarct located slightly apart from the main ischemic core; this area was selected as representative because of its low background signal. Scale bar = 100 μm. (E) GFP-positive transplanted severe-hypo-OPCs were detected without additional anti-GFP staining (No staining), retained PDGFRα expression, and some were phagocytosed by IBA1-positive microglia. Scale bar = 30 μm. (F) GFP-positive transplanted severe-hypo-OPCs did not express oligodendrocyte maturation markers O4 and GST-π. Scale bar = 30 μm. Abbreviations: Log1p Nor Expr, Log1p-transformed normalized expression; Sc AT, Subcutaneous Adipose Tissue; Vis AT, Visceral Adipose Tissue.

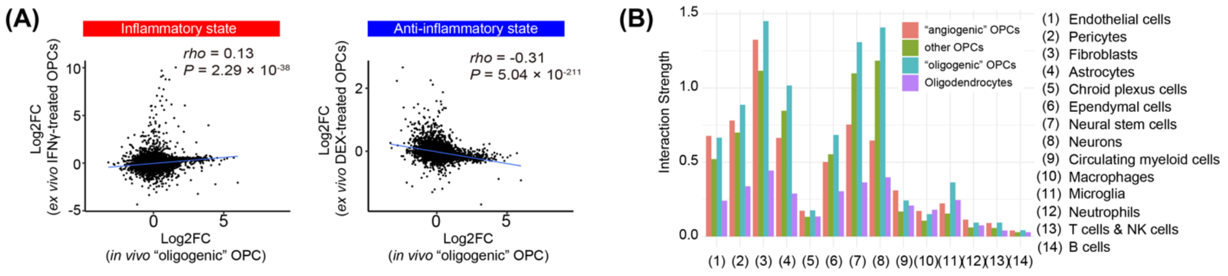

Fig. S5. The inflammatory state and cell-cell interactions may influence the transcriptomic characteristics of *in vivo* "oligogenic" oligodendrocyte precursor cells (OPCs). (A) Correlation analysis revealed significant positive correlations of $\log_2$ fold changes ($\log_2$FC) between in vivo "oligogenic" OPCs and ex vivo interferon-γ (IFN-γ)-treated OPCs, as well as notable negative correlations of log2FC between *in vivo* "oligogenic" OPCs and *ex vivo* dexamethasone (DEX)-treated OPCs. (B) Cell-cell interaction analysis revealed enhanced interactions between "oligogenic" OPCs and various cell types. Abbreviations: *rho*, Spearman's rank correlation coefficient.
