# Supplementary material for: Characterizing hypoxia-orchestrated post-stroke changes in oligodendrocyte precursor cells for optimized cell therapy
Source: Stem Cell Reports. 2025 Oct 30;20(11):102687. doi: 10.1016/j.stemcr.2025.102687 (PMC12790735; doi:10.1016/j.stemcr.2025.102687)
Supplement: Document S2. Article plus supplemental information [file mmc2.pdf]

Stem Cell Reports
Article
OPEN ACCESS

Characterizing hypoxia-orchestrated post-stroke changes in oligodendrocyte precursor cells for optimized cell therapy

Yasuhiro Kuwata,[1,5] Ken Yasuda,[1,5,*] Kazuto Tsukita,[1,2,3,5,*] Akihiro Kikuya,[1] Naoki Takayama,[4] Narufumi Yanagida,[1] Kimitoshi Kimura,[1] Ryosuke Takahashi,[1] Riki Matsumoto,[1] and Takakuni Maki[1,6,*]
[1]Department of Neurology, Kyoto University Graduate School of Medicine, Sakyo-ku, Kyoto 606-8507, Japan
[2]Advanced Comprehensive Research Organization, Teikyo University, Itabashi-ku, Tokyo 173-0003, Japan
[3]Division of Sleep Medicine, Kansai Electric Power Medical Research Institute, Fukushima-ku, Osaka 553-0003, Japan
[4]Department of Neurosurgery, Kyoto University Graduate School of Medicine, Sakyo-ku, Kyoto 606-8507, Japan
[5]These authors contributed equally
[6]Lead contact
*Correspondence: kenyasud@kuhp.kyoto-u.ac.jp (K.Y.), kazusan@kuhp.kyoto-u.ac.jp (K.T.), harutoma@kuhp.kyoto-u.ac.jp (T.M.)
https://doi.org/10.1016/j.stemcr.2025.102687

SUMMARY

Oligodendrocyte precursor cells (OPCs) are highly adaptable, engaging in diverse functions beyond myelination. However, how OPCs adjust their roles after ischemic stroke and contribute to recovery remains largely unknown. To address this gap, we constructed a "transient middle cerebral artery occlusion (tMCAO) atlas" by integrating mouse single-cell RNA sequencing (scRNA-seq) datasets and combined it with *ex vivo* OPC cultures and *in vivo* cell transplantation experiments. This approach revealed the emergence of "angiogenic" OPCs in the subacute phase and "oligogenic" OPCs in the chronic phase, driven by distinct levels of hypoxia—severe hypoxia inducing angiogenic OPCs and mild hypoxia promoting oligogenic OPCs. *Ex vivo*, severe hypoxic preconditioning faithfully induced angiogenic OPCs, and their intravenous transplantation enhanced angiogenesis and improved recovery in tMCAO mice. These findings highlight "oxygen tone" as a key regulator of OPC dynamic adaptation after ischemic stroke, offering a promising strategy to harness OPCs for stroke cell therapy.

INTRODUCTION

Oligodendrocyte precursor cells (OPCs) originate during embryogenesis and persist as resident cells in the adult brain parenchyma (Akay et al., 2021; Bergles and Richardson, 2015). Initially generated in the subventricular zones of the embryonic neural tube, OPCs migrate throughout the brain and spinal cord (Tsai et al., 2016), continuing to proliferate and develop into oligodendrocytes during postnatal development (Akay et al., 2021; Bergles and Richardson, 2015). While the rate of oligodendrocyte production significantly declines as the brain matures, OPCs remain abundant in the adult brain (Akay et al., 2021; Bergles and Richardson, 2015). OPCs' primary role is to differentiate into oligodendrocytes and contribute to myelination (Akay et al., 2021; Bergles and Richardson, 2015); however, it is increasingly recognized that OPCs actively modify their characteristics in response to the surrounding environment, participating in various aspects of brain development, structure, and function through interactions with other cells (Akay et al., 2021; Xiao and Czopka, 2023).

Among various environmental cues influencing OPCs, growing evidence suggests that they utilize hypoxia-inducible factor 1α (HIF-1α), a key regulator of cellular responses to hypoxia, to shift toward an angiogenic phenotype during embryonic development (Yuen et al., 2014). In the fetal brain, extremely low oxygen levels (partial pressure of oxygen ≤ 7.60 mmHg, equivalent to a fraction of inspiratory oxygen [FiO2] ≤ 1.00%) render HIF activity indispensable for brain development (Zhang et al., 2011). During embryonic development, HIF-1α-instructed OPCs inhibit their maturation into oligodendrocytes by upregulating WNT signaling in an autocrine manner and/or by suppressing *Sox10* expression via non-canonical HIF-1α targets (Allan et al., 2021; Yuen et al., 2014). At the same time, they promote angiogenesis in a paracrine manner by activating WNT and/or vascular endothelial growth factor receptor signaling in endothelial cells (Allan et al., 2021; Minocha et al., 2015; Yuen et al., 2014). Notably, autocrine activation of WNT signaling in OPCs upregulates *Cxcr4*, facilitating OPC association with endothelial cells through the CXCR4-CXCL12 axis (Fancy et al., 2009; 2014; Tsai et al., 2016). Thus, a key role of OPCs in embryonic brain development is to associate with vasculature and promote angiogenesis, driven by severe hypoxia in the fetal brain and coordinated by HIF-1α, WNT signaling, and *Cxcr4*.

Importantly, cerebral ischemia is another condition where hypoxia-driven cellular modulation becomes prominent (Baranova et al., 2007). Interestingly, although prolonged extreme hypoxia leads to cell death, sub-lethal hypoxic conditions can shift brain tissue toward greater resilience against cerebral ischemia (Rybnikova et al., 2022). Although the mechanisms of hypoxia-induced tolerance to ischemic stroke have mainly focused on neuronal cells, it has also been reported that neuronal changes alone do not fully explain the observed resilience (He et al., 2021). This highlights the important role of other cell types

Published by Elsevier Inc. on behalf of International Society for Stem Cell Research.
This is an open access article under the CC BY license (http://creativecommons.org/licenses/by/4.0/).

within the neurovascular unit, including OPCs, in stroke pathophysiology (Tiedt et al., 2022; Kishida et al., 2019; Okazaki et al., 2019; Hase et al., 2022).

Current acute ischemic stroke therapies primarily aim to rapidly restore cerebral blood flow to minimize damage (Chamorro et al., 2021). While their success depends on how quickly and effectively blood flow is restored, complete recovery is not always achievable in all patients, underscoring the need for additional therapies to provide "neuroprotection" (Chamorro et al., 2021). Among potential therapeutic approaches, stem cell transplantation is particularly promising, as it can provide the necessary cellular components that are otherwise insufficient in the post-stroke brain (Anthony et al., 2022). Although the potential of stem cell transplantation for ischemic stroke treatment is unquestionable (Anthony et al., 2022), its clinical translation to humans remains challenging (Houkin et al., 2024), highlighting the unmet need for further refinement of cell-based therapies. To this end, we propose two key steps: (1) identifying specific cells that inherently protect ischemic tissue but are insufficient on their own and (2) understanding their adaptive responses (Kakae et al., 2023; Kumar Podder et al., 2024). Following ischemic stroke, the brain undergoes dynamic biological changes driven by both local interactions within the neurovascular unit and systemic metabolic shifts (Hosoki et al., 2020; Tiedt et al., 2022). Therefore, a thorough understanding of these biological events is crucial to identifying protective responses that should be supported. In this context, a promising strategy to improve stem cell transplantation is to modify these cells *ex vivo* to mimic *in vivo* adaptations, thereby enhancing their protective functions and optimizing therapeutic outcomes.

Here, we focused on OPCs for two reasons: (1) under physiological conditions, only a limited number of OPCs migrate to ischemic regions (Wang et al., 2022); and (2) our previous studies demonstrated that *ex vivo* OPCs preconditioned with severe hypoxia secrete a variety of pro-angiogenic factors (Kishida et al., 2019). These findings suggest that hypoxia-induced phenotypic changes in OPCs can enhance ischemic tolerance in brain tissue, though their natural response remains suboptimal.

In this study, we first compiled and analyzed publicly available single-cell RNA sequencing (scRNA-seq) datasets to construct a "transient middle cerebral artery occlusion (tMCAO) atlas" and profile OPC transcriptional changes during ischemic stroke. We chose tMCAO as the model for acute ischemic stroke as it is a well-established and widely used model (Frazier et al., 2023; Kim et al., 2022; Nakahashi-Oda et al., 2021; Shi et al., 2021a; 2021b; Wu et al., 2023; Zeng et al., 2023; Zheng et al., 2022). This model induces ischemic injury in the ipsilateral cortex, striatum (including the caudoputamen), and adjacent white matter, thereby closely reproducing the distribution of infarcts typically observed in patients with middle cerebral artery territory stroke. Importantly, tMCAO allows reperfusion, which is highly relevant to current clinical practice involving thrombolysis and thrombectomy. Using the tMCAO atlas, we found that a subset of OPCs altered their transcriptome in the subacute stage after tMCAO (day 3), adopting a distinct hypoxia-induced, HIF-1α-driven "angiogenic" transcriptional profile that mirrors OPC characteristics reported in embryonic brain development (Yuen et al., 2014). In contrast, in the chronic stage after tMCAO (day 14), OPCs transitioned into a distinct "oligogenic" transcriptional profile. Importantly, *ex vivo*, we successfully induced OPCs to acquire transcriptomic characteristics similar to those of *in vivo* "angiogenic" OPCs by utilizing severe hypoxia preconditioning. Notably, intravenous transplantation of these cells efficiently promoted angiogenesis, reduced infarct volume, and ameliorated functional decline after tMCAO. Finally, we demonstrated that varying oxygen levels critically influence OPC maturation *ex vivo*, with mild hypoxia optimally promoting OPC maturation and, at least in part, contributing to the emergence of *in vivo* "oligogenic" OPCs. These findings underscore the crucial role of "oxygen tone" in shaping temporally dynamic reparative OPC phenotypes and highlight the therapeutic potential of hypoxia-preconditioned OPCs for enhancing post-stroke recovery.

RESULTS

Creation of the tMCAO atlas

We systematically searched the BioProject database to identify scRNA-seq datasets that investigated post-tMCAO transcriptional changes and utilized the droplet-based 10× Genomics Chromium approach (see Methods) (Frazier et al., 2023; Kim et al., 2022; Nakahashi-Oda et al., 2021; Shi et al., 2021a; 2021b; Wu et al., 2023; Zeng et al., 2023; Zheng et al., 2022). To minimize batch effects across studies as much as possible, we downloaded raw FASTQ data and processed all datasets using the same pipeline for mapping, followed by uniform and stringent quality control according to single-cell best practices (see Methods) (Heumos et al., 2023). For our meta-analysis, we analyzed scRNA-seq datasets derived primarily from the ipsilateral hemisphere after tMCAO, encompassing both the infarct core and peri-infarct regions. Contralateral hemispheres from tMCAO mice were excluded, and control datasets were obtained from the ipsilateral hemisphere of sham-operated mice. Ultimately, we included 21 samples from 6 studies that provided sufficient OPCs to construct the tMCAO atlas (Table S1). After quality control, our tMCAO atlas comprised 106,905 cells from

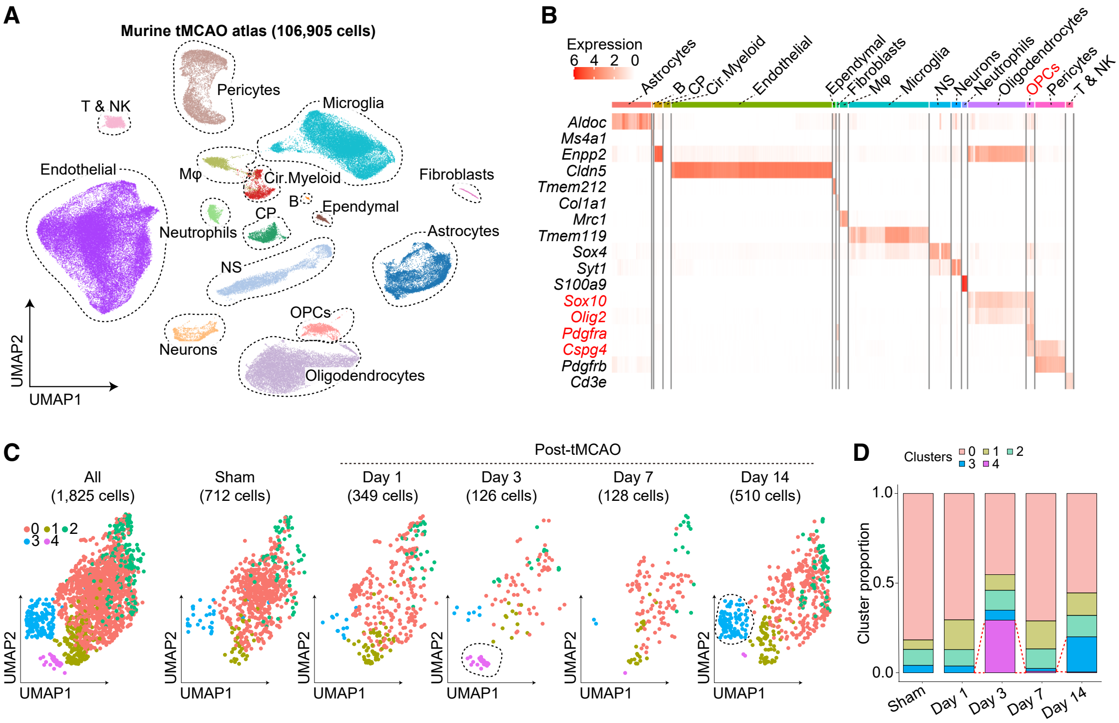

Figure 1. Murine transient middle cerebral artery occlusion atlas identifies distinct subclusters of oligodendrocyte precursor cells
(A) The transient middle cerebral artery occlusion (tMCAO) atlas comprises 106,905 cells from 21 oligodendrocyte precursor cell (OPC)-containing samples across six studies.
(B) OPCs were extracted based on the expression of *Sox10*, *Olig2*, *Pdgfra*, and *Cspg4* genes.
(C) Dimensional reduction using uniform manifold approximation and projection (UMAP) reveals distinct OPC clusters, including cluster 4 at day 3 post tMCAO and cluster 3 at day 14 post tMCAO.
(D) The proportion of cluster 4 increases on day 3 post tMCAO, whereas cluster 3 becomes more prominent on day 14 post tMCAO. Abbreviations: B, B cells; CP, choroid plexus cells; Cir.Myeloid, circulating myeloid cells; Endothelial, endothelial cells; Ependymal, ependymal cells; Mφ, macrophages; NP, neural progenitor cells; T & NK, T cells and NK cells.

sham-operated, 1-day, 3-day, 7-day, and 14-day post-tMCAO groups (Figures 1A and 1B).

Transcriptomic characterization of angiogenic OPCs on day 3 and oligogenic OPCs on day 14 after tMCAO

We identified OPCs based on the expression of *Pdgfrα*, *Cspg4*, *Sox10*, and *Olig2* genes and primarily focused on OPCs for subsequent analyses (Figure 1B). Notably, we noted two distinct clusters: one that increased on day 3 after tMCAO (cluster 4) and another that increased on day 14 after tMCAO (cluster 3) (Figures 1C and 1D). Importantly, neither cluster 4 nor cluster 3 expressed genes specific to non-OPC cell types (Figure S1A), and both were consistently detected in samples at 3 and 14 days post tMCAO, respectively (Figure S1B), indicating that these clusters likely represent significant time-course-specific biological changes rather than batch effects.

To investigate the overall functional properties of these distinct clusters, we conducted gene set scoring analysis using pathways retrieved from the Molecular Signatures Database (Castanza et al., 2023), including Hallmark gene sets, Gene Ontology gene sets, and WIKI pathways gene sets. Additionally, to infer transcription factor activity, we used the CollecTRI database for transcription factor regulons (Müller-Dott et al., 2023). Finally, to examine OPC-specific functions, we manually curated gene sets, including OPC migration/myelination-associated gene sets (Hamanaka et al., 2023) and OPC-specific HIF-1α target gene sets (Allan et al., 2021; Hamanaka et al., 2023). We also extracted signature of WNT-activated OPCs via reanalyzing microarray datasets of mouse WNT-activated OPCs from Olig2cre-DA-cat mice (Fancy et al., 2009; 2014; Tsai et al., 2016), in which dominant-active β-catenin allows constitutive activation of the WNT pathway only in oligodendrocyte lineage cells.

These analyses revealed that OPCs in cluster 4, a distinct population present 3 days post-tMCAO, specifically upregulate HIF-1α activity, as evidenced by increased expression

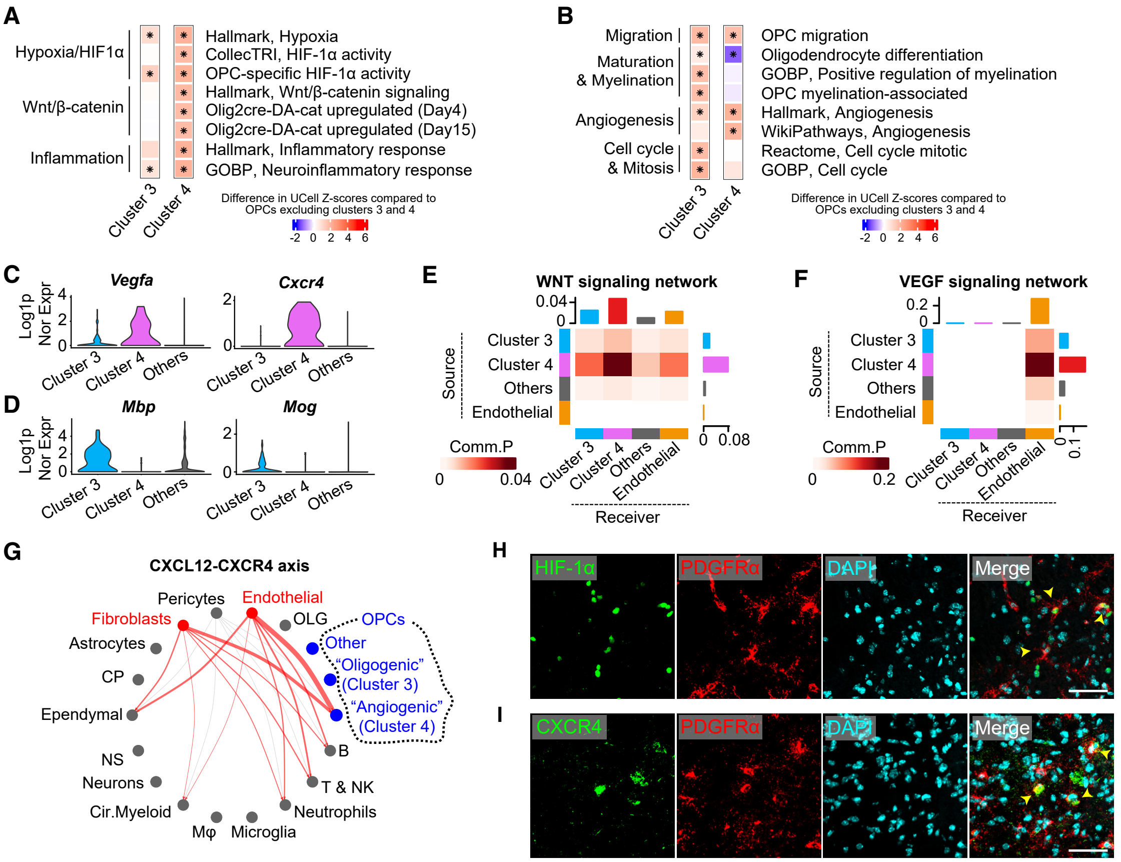

Figure 2. Bioinformatic analyses predict the functions of distinct OPC subclusters as angiogenic and oligogenic

(A) Gene set scoring analyses revealed upregulated pathways in OPC subclusters compared to other OPCs outside clusters 3 and 4. Mann-Whitney U test with multiple comparisons adjusted using the Benjamini & Hochberg method, $p < 0.05$.

(B) Gene set scoring analyses identified upregulated OPC-relevant functional properties in OPC subclusters compared to other OPCs outside clusters 3 and 4. Mann-Whitney U test with multiple comparisons adjusted using the Benjamini and Hochberg method, $p < 0.05$.

(C) *Vegfa* and Cxcr4 were specifically upregulated in cluster 4 OPCs.

(D) *Mog* and *Mbp* were specifically upregulated in cluster 3 OPCs.

(E and F) Cell-cell interaction analyses revealed that cluster 4 OPCs act as critical signaling sources in the WNT and VEGF signaling networks, with endothelial cells serving as key recipients.

(G) The CXCL12-CXCR4 axis was specifically upregulated from endothelial cells and fibroblasts to cluster 4 OPCs.

(H and I) Immunohistochemistry demonstrated upregulated HIF-1α activity and CXCR4 expression in subsets of OPCs accumulating at the ischemic border. Scale bars, 50 μm.

of both canonical and OPC-specific HIF-1α target genes (Figure 2A), including marked upregulation of the *Vegfa* gene (Figure 2C). Consistent with previous reports on HIF-1α-instructed OPCs (Yuen et al., 2014), this cluster also exhibits heightened WNT pathway activity (Figure 2A), with a drastic increase in *Cxcr4* expression (Figure 2C) (Tsai et al., 2016). Collectively, these findings collectively indicate that severe hypoxia plays a key role in inducing cluster 4 of OPCs.

Notably, cluster 4 also exhibits specific upregulation of genes involved in the inflammatory response (Figure 2A) and is predicted to be influenced by cytokines, such as interleukin-1β (IL-1β), tumor necrosis factor alpha (TNF-α), and interferon-γ (IFN-γ), which have been widely implicated in the pathological processes during the acute phase of ischemic stroke (Doll et al., 2014; Li et al., 2001) (Figure S1C). This suggests that inflammation associated with the acute phase of ischemic stroke may also modify the characteristics of this cluster.

Functionally, cluster 4 upregulated genes associated with OPC migration (Figure 2B). In line with the characteristics of HIF-1α-instructed OPCs observed in the developing brain (Allan et al., 2021; Yuen et al., 2014), OPCs in cluster 4 appeared to halt gene programs related to oligodendrocyte differentiation and subsequent myelination (Figure 2B), as evidenced by the reduced expression of

myelination-associated *Mbp* and *Mog* genes (Figure 2D). Instead, the defining feature of OPCs in this cluster appears to be their active involvement in angiogenesis (Figure 2B). Consistently, cell-cell interaction analyses revealed that OPCs in this cluster closely associate with endothelial cells, acting as a signaling source through the WNT and vascular endothelial growth factor (VEGF) pathways (Figures 2E and 2F), both of which are essential for OPC-driven angiogenesis in the developing brain (Allan et al., 2021; Yuen et al., 2014). Conversely, endothelial cells appear to attract OPCs in cluster 4 via the CXCL12-CXCR4 axis (Figure 2G), a key pathway for the close anatomical association between OPCs and endothelial cells in the developing brain (Tsai et al., 2016). It is also noteworthy that, even in comparison to other cell types, OPCs in cluster 4 represent a significant source of *Vegfa* at 3 days after tMCAO (Figure S1E), alongside astrocytes and fibroblasts, and are the primary source of *Wnt7a* at 3 days after tMCAO (Figure S1F), both of which are key factors in post-stroke angiogenesis (Hu et al., 2024). Altogether, we concluded that cluster 4 represents the OPCs primarily instructed by HIF-1α, resembling the angiogenesis-inducing OPCs previously characterized in the developing brain (Allan et al., 2021; Minocha et al., 2015; Tsai et al., 2016; Yuen et al., 2014), with additional functional modulation likely influenced by inflammation. We therefore annotated cluster 4 as angiogenic OPCs.

To validate the presence and investigate the spatial distribution of angiogenic OPCs, we next conducted immunofluorescence analysis 3–5 days post tMCAO. Nuclear HIF-1α signals were observed in a subset of platelet-derived growth factor receptor alpha (PDGFRα)-positive cells exhibiting morphological characteristics consistent with OPCs at the peri-infarct region (Figure 2H). Additionally, in the peri-infarct cortex, we identified cells double-positive for VEGFA and OLIG2 (Figure S2A), as well as cells double-positive for CXCR4 and PDGFRα (Figure 2I)—a combination that effectively distinguishes angiogenic OPCs from other cell types (Figures S1E, S1G, and S1H)—but not in the ischemic core or in the peri-infarct corpus callosum with its adjacent regions (Figure S2B).

Interestingly, OPCs in cluster 3, a distinct population observed 14 days post tMCAO, exhibit functional characteristics that are markedly different from the angiogenic OPCs seen at 3 days post tMCAO. This cluster appears to be only mildly affected by hypoxia, moderately upregulates OPC-specific HIF-1α target genes, and slightly increases the expression of angiogenesis-related genes (Figure 2A). However, its defining feature is the elevated expression of genes involved in oligodendrocyte differentiation and myelination (Figure 2B), such as *Mbp* and *Mog* genes (Figure 2D), accompanied by pronounced cellular renewal through cell division (Figure 2B). Prominently, *Myrf*, a key transcription factor for oligodendrocyte myelination and myelin maintenance (Emery et al., 2009; Qian et al., 2021), and *Bcas1*, a marker of the active phase of oligodendrocyte generation and myelination, are specifically upregulated in OPCs in cluster 3 (Fard et al., 2017) (Figure S1D). Additionally, these cells are predicted to be influenced not only by acute-phase cytokines like IL-1β, TNF-α, and IFN-γ but also by cytokines more associated with the chronic phase, such as IL-10 and transforming growth factor β (Doll et al., 2014; Hu et al., 2024) (Figure S1C). Based on these observations, we annotated cluster 3 as oligogenic OPCs, which are likely to play an integral role in remyelination following the acute stage of ischemic stroke. This role appears to be shaped by a milder hypoxic environment than that of angiogenic OPCs, as well as the influence of chronic-phase cytokines.

We next turned to immunohistochemical analyses to confirm the presence of oligogenic OPCs *in vivo* and assess their spatial distribution 14 days post tMCAO. In contrast to angiogenic OPCs, BCAS1$^+$OLIG2$^+$ oligogenic OPCs were more prominently increased in the peri-infarct corpus callosum with its adjacent regions, relative to the peri-infarct cortex, at day 14 post tMCAO (Figures S2C–S2E). These results confirmed the presence of the identified OPC subtypes and further indicated that the temporal transitions of OPC phenotypes after ischemic stroke are coupled with distinct spatial distributions, with angiogenic OPCs predominantly localized to peri-infarct cortical regions at day 3 and oligogenic OPCs enriched in peri-infarct white matter at day 14.

Interestingly, trajectory analysis predicted two distinct differentiation trajectories (Figures S2F and S2G). The first follows an angiogenic trajectory, culminating in angiogenic OPCs (Figure S2H), while the second represents an oligogenic trajectory, leading to oligogenic OPCs (Figure S2I). In both trajectories, key genes characteristic of each OPC population show progressively increased expression as differentiation advances (Figure S2J).

Ex vivo induction of HIF-1α-instructed angiogenic OPCs via severe hypoxic preconditioning

Encouraged by the *in vivo* detection of HIF-1α-instructed angiogenic OPCs after tMCAO, we reanalyzed bulk RNA sequencing (RNA-seq) data of OPCs subjected to severe hypoxic preconditioning *ex vivo* (severe-hypo-OPCs), previously generated by our group (Figure 3A) (Kishida et al., 2019).

First, we aimed to confirm that our *ex vivo* OPC cultures faithfully reflect the transcriptional characteristics of *in vivo* OPCs in the murine brain. Analysis of scRNA-seq data from murine oligodendrocyte lineage cells (Marques et al., 2016) demonstrated that our *ex vivo* OPCs closely resemble the transcriptional profile of *in vivo* OPCs, although some cells exhibit signs of differentiation into committed

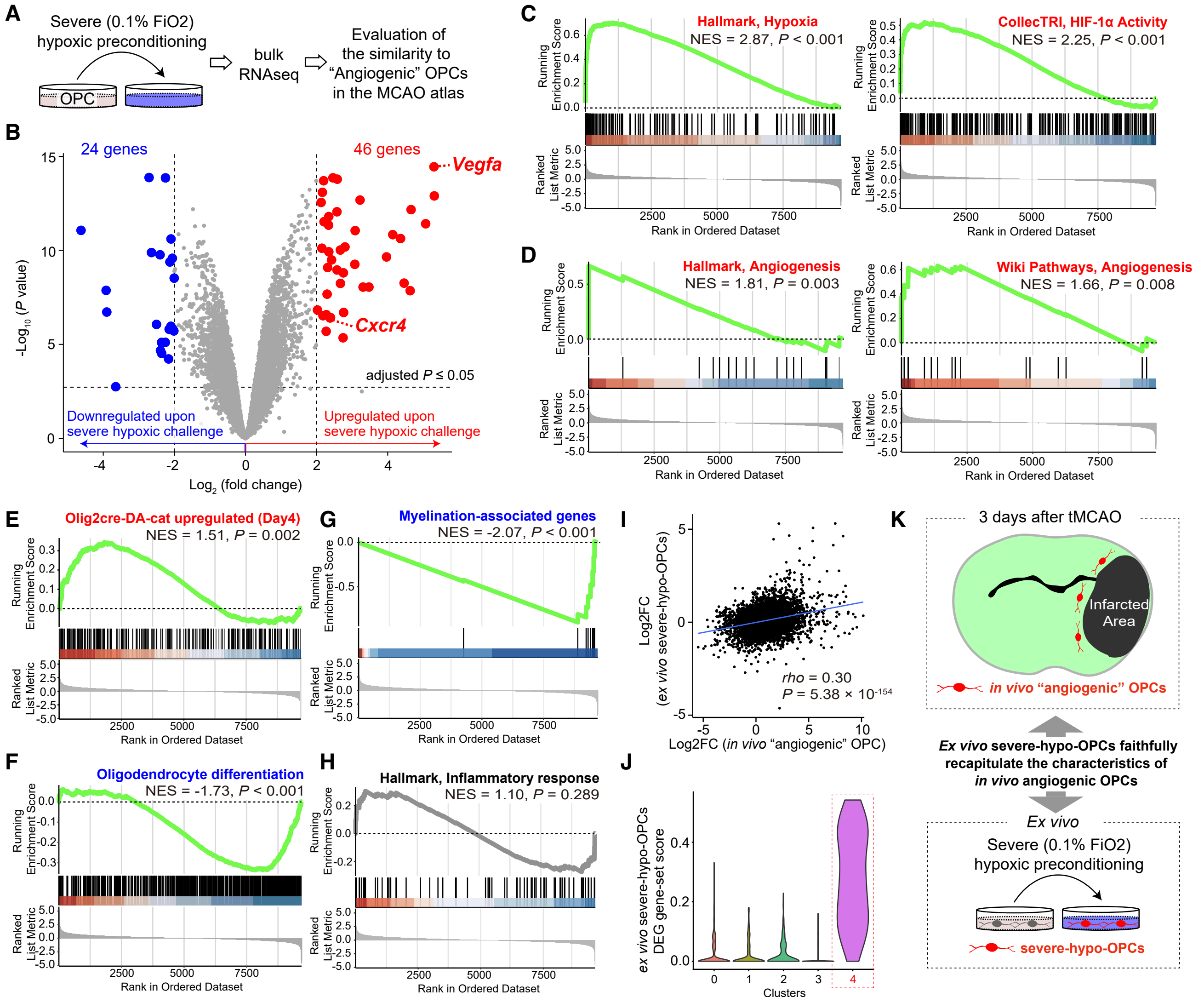

Figure 3. *Ex vivo* OPCs acquire functional characteristics similar to *in vivo* angiogenic OPCs following severe hypoxic preconditioning

(A and B) Bulk RNA-seq-based differentially expressed gene (DEG) analysis revealed marked transcriptional changes in *ex vivo* severe hypoxia-preconditioned OPCs (severe-hypo-OPCs), including significant upregulation of *Vegfa* and *Cxcr4* and hallmark genes of *in vivo* angiogenic OPCs. Red points indicate significantly different genes identified using a linear model with multiple comparisons adjusted by the Benjamini and Hochberg method, with significance set at $p < 0.05$ and $\log_2$ fold change >2. $n = 3$ (control, biological replicates) and $n = 3$ (0.1%, biological replicates).

(C–H) Gene set enrichment analysis (GSEA) revealed increased hypoxia and HIF-1α pathway activities (C), angiogenesis pathway activities (D), and WNT pathway activities (E); decreased OPC differentiation toward oligodendrocytes (F); decreased myelination-associated gene expression (G) in severe-hypo-OPCs, faithfully mirroring the functional characteristics of *in vivo* angiogenic OPCs. However, no upregulation of the inflammatory response was detected (H).

(I) Correlation analysis revealed a significant positive correlation of $\log_2$ fold changes ($\log_2$FC) between *in vivo* angiogenic OPCs and *ex vivo* severe-hypo-OPCs.

(J) Gene set scores calculated from DEGs in *ex vivo* severe-hypo-OPCs were specifically upregulated in cluster 4 OPCs in the transient middle cerebral artery occlusion (tMCAO) atlas.

(K) Illustration showing that *ex vivo* severe-hypo-OPCs faithfully recapitulate the characteristics of *in vivo* angiogenic OPCs, which emerge at the peri-infarct region 3 days post tMCAO. FiO_2, fraction of inspiratory oxygen; NES, normalized enrichment score; Hallmark, Hallmark gene sets; CollecTRI, CollecTRI database; DA-cat, dominant-active β-catenin; GOBP, Gene Ontology Biological Process; WikiPathways; *rho*, Spearman's rank correlation coefficient.

oligodendrocyte precursors and newly formed oligodendrocytes (Figure S3A). These findings validate our *ex vivo* OPC cultures as a suitable model for evaluating functional changes in OPCs *in vivo*.

Crucially, differentially expressed gene (DEG) analysis revealed substantial transcriptional changes within severe-hypo-OPCs compared with standard OPCs, including significant upregulation of *Vegfa* and *Cxcr4* (Figure 3B). As expected, gene set enrichment analysis (GSEA) suggested that hypoxia-induced changes underlie these marked transcriptional alterations, and HIF-1α activation played an orchestrating role (Figure 3C). Notably, severe-hypo-OPCs dramatically upregulated angiogenesis activity (Figure 3D) and WNT pathway activity (Figure 3E), along with apparent downregulation of genes associated with oligodendrocyte differentiation and subsequent myelination (Figures 3F and 3G), mirroring the functional characteristics of *in vivo* angiogenic OPCs detected 3 days post tMCAO. Although we did not observe upregulation of inflammatory response genes in severe-hypo-OPCs (Figure 3H), correlation analyses between the log2-fold changes of DEGs in these *ex vivo* severe-hypo-OPCs and those in the *in vivo* angiogenic OPCs revealed significant positive correlations (*rho* = 0.30, Figure 3I). Given that cross-platform correlations between scRNA-seq and bulk RNA-seq $\log_2$ fold changes are typically around *rho* ≈ 0.6 even for identical samples—primarily due to the substantial difference in sequencing depth between the two platforms (Liu et al., 2023)—the observed correlation of *rho* = 0.30 should be interpreted as biologically relevant. Furthermore, the gene set score calculated from DEGs in *ex vivo* severe-hypo-OPCs confirmed a marked increase in the coordinated upregulation of these DEGs in *in vivo* angiogenic OPCs (Figure 3J).

To further investigate the contribution of the inflammatory response in shaping *in vivo* angiogenic OPCs, we reanalyzed bulk RNA-seq data from OPCs treated with IFN-γ (pro-inflammatory) or dexamethasone (DEX; anti-inflammatory) (Meijer et al., 2022). In both conditions, there were no changes in the expression of *Vegfa* and *Cxcr4* (Figures S3B and S3C), indicating that inflammation is unlikely to be the main driver of gene expression changes in *in vivo* angiogenic OPCs. Nevertheless, IFN-γ treatment did suppress the expression of myelination-associated genes (Figures S3D and S3F), and the gene expression changes observed in *ex vivo* IFN-γ- and DEX-treated OPCs showed weak positive and negative correlations, respectively, with those observed in *in vivo* angiogenic OPCs (Figures S3E and S3G). Collectively, we concluded that *ex vivo* severe-hypo-OPCs faithfully replicate the functional characteristics of *in vivo* angiogenic OPCs detected 3 days post tMCAO, although additional modifications influenced by inflammatory responses may further alter the properties of *in vivo* angiogenic OPCs (Figure 3K).

Transplantation of severe-hypo-OPCs effectively ameliorated tMCAO outcome through increasing angiogenesis

Considering the well-known benefits of angiogenesis (Fang et al., 2023; Kanazawa et al., 2019; Krupinski et al., 1994), we hypothesized that *in vivo* angiogenic OPCs play a protective role. Encouraged by the finding that *ex vivo* severe-hypo-OPCs exhibit similar functional characteristics to *in vivo* angiogenic OPCs, we transplanted these severe-hypo-OPCs and compared their efficacy with standard OPCs. The angiogenic OPCs emerge at 3 days post tMCAO, and angiogenesis is reported to be detectable around 3–4 days after tMCAO (Fang et al., 2023; Kanazawa et al., 2019; Krupinski et al., 1994); therefore, we chose to administer severe-hypo-OPCs at 3 days post tMCAO. While previous OPC transplantation studies have primarily employed intracranial injection (Chen et al., 2015; Li et al., 2021; Wang et al., 2022), we opted for intravenous transplantation due to the following reasons: (1) severe-hypo-OPCs are likely to interact readily with brain endothelial cells and fibroblasts through the CXCR4-CXCL12 axis (Figure 2G); (2) the tMCAO atlas indicates upregulation of *Cxcl12* expression in brain endothelial cells and fibroblasts at 3 days post tMCAO (Figure S4A); and (3) brain endothelial cells express *Cxcl12* at higher levels than endothelial cells from other organs, as shown by public scRNA-seq data on multi-organ endothelial cells (Figures S4B and S4C) (Bondareva et al., 2022). Importantly, intravenous injection also has far greater clinical translation potential compared to intracranial injection.

We administered a retro-orbital injection of 7.5×10^5 CAG-enhanced green fluorescent protein (EGFP)-tagged severe-hypo-OPCs suspended in 5 μL PBS (severe-hypo-OPC group), CAG-EGFP-tagged standard OPCs suspended in 5 μL PBS (standard OPC group), or 5 μL PBS alone (control group) on day 3 post tMCAO. Neurological function was assessed over time, and on day 14, we measured the percentage of infarcted areas in each mouse, a commonly used endpoint around the time when brain repair processes typically activate (Clarkson et al., 2010; Shi et al., 2021a) (Figure 4A). At 14 days after transplantation, co-staining with microtubule-associated protein-2 (MAP-2) revealed that GFP+-transplanted OPCs were distributed not only within the ischemic core and border but also extended into the peri-infarct penumbra, indicating that the transplanted OPCs survived and remained engrafted in the host tissue at day 14 (Figure S4D). Neurobehavioral tests revealed that the severe-hypo-OPC group exhibited the greatest recovery compared to the standard OPC and control groups. This was evident both in modified neurological severity scores (mNSS) (at day 14: −2.17 ± 0.41 [severe-hypo-OPC] vs. −1.33 ± 0.52 [standard OPC] vs. 0.00 ± 0.60 [control]) and in the Rotarod test (time to fall

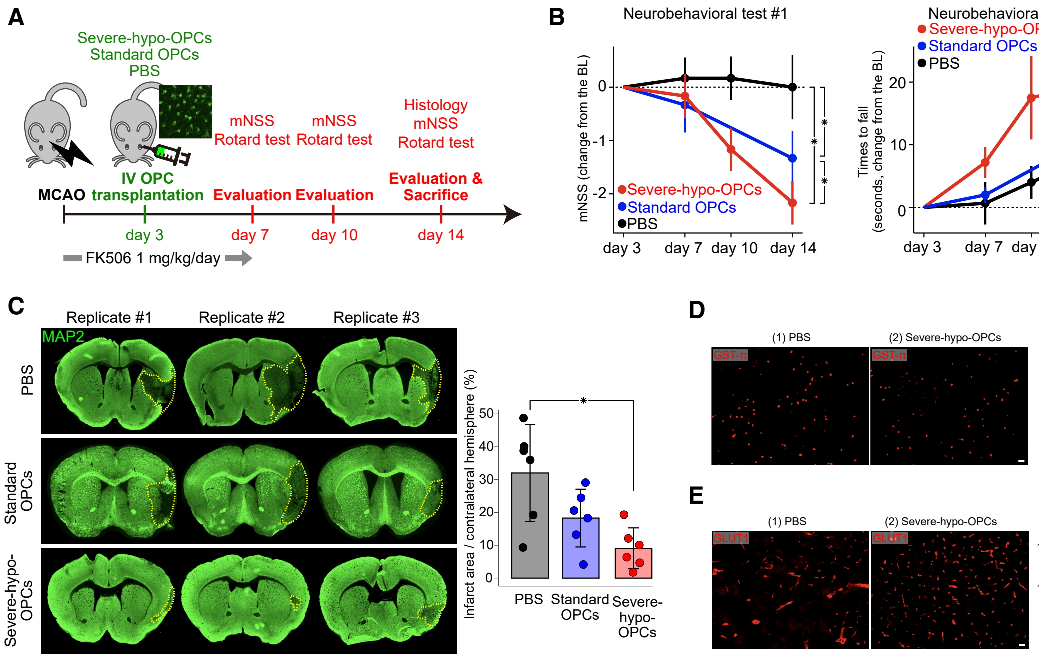

Figure 4. Intravenous transplantation of severe-hypo-OPCs facilitates post-tMCAO recovery by promoting angiogenesis

(A) Intravenous (i.v.) transplantation of OPCs (severe-hypo-OPCs, standard OPCs, and PBS) was performed on day 3 post tMCAO. Serial neurobehavioral assessments and histological quantifications of the infarcted area were subsequently conducted.

(B) Neurobehavioral tests confirmed accelerated recovery in the severe-hypo-OPC group. Circles indicate the mean, and error bars represent the standard deviation. Mann-Whitney U test with multiple comparisons adjusted using the Benjamini and Hochberg method, $p < 0.05$; $n = 12$ (control, biological replicates), $n = 6$ (severe-hypo-OPC, biological replicates), $n = 6$ (standard OPC, biological replicates).

(C) MAP-2 immunofluorescence-based quantification of the infarcted area showed a reduced infarct size in the severe-hypo-OPC group. The yellow dashed line indicates the infarcted area. The tops of the bars represent the mean, and error bars indicate the standard deviation. Mann-Whitney U test with multiple comparisons adjusted using the Benjamini and Hochberg method, $p < 0.05$; $n = 6$ (control, biological replicates), $n = 6$ (severe-hypo-OPC, biological replicates), $n = 6$ (standard OPC, biological replicates).

(D) The number of GST-π-positive cells remained unchanged between the control (phosphate-buffered saline [PBS]) and severe-hypo-OPC groups. The tops of the bars indicate the mean, and the error bars indicate the standard deviation. Scale bars, 50 μm. $n = 3$ (severe-hypo-OPC), $n = 3$ (standard OPC).

(E) The number of branches in blood vessels was significantly higher in mice intravenously injected with severe-hypo-OPCs. The tops of the bars indicate the mean, and the error bars indicate the standard deviation. Scale bars, 50 μm. Mann-Whitney U test, $^*p < 0.05$; $n = 3$ (severe-hypo-OPC), $n = 3$ (standard OPC).

at day 14: 19.83 ± 4.49 [severe-hypo-OPC] vs. 11.33 ± 2.34 [standard OPC] vs. 8.50 ± 3.34 [control]) (Figure 4B). Critically, the severe-hypo-OPC group exhibited the smallest infarcted area among the three groups (% infarcted area at day 14: 10.57 ± 6.36 [severe-hypo-OPC] vs. 18.28 ± 8.80 [standard OPC] vs. 32.02 ± 14.74 [control]).

Immunofluorescence analyses revealed that transplanted severe-hypo-OPCs expressed PDGFRα (Figure S4E) but did not express O4 or glutathione-S transferase (GST)-π (Figure S4F), suggesting that they remained immature and did not differentiate into oligodendrocytes. Approximately half of the transplanted severe-hypo-OPCs colocalized with ionized calcium-binding adaptor molecule-1 (Iba-1)-positive cells (Figure S4E), indicating that they were phagocytosed by microglia. Regarding the therapeutic mechanism of transplanted severe-hypo-OPCs, immunofluorescence showed no change in the number of GST-π-positive cells (Figure 4D), but vascular density and branching were significantly increased following severe-hypo-OPC transplantation (Figure 4E). Thus, these findings collectively indicated that intravenous transplantation of severe-hypo-OPCs efficiently reduced infarct size and improved stroke outcomes primarily by promoting angiogenesis rather than oligodendrogenesis.

Oxygen tone contributes to regulating OPC phenotypic change during the tMCAO time course

Although the hypoxic response in *in vivo* oligogenic OPCs appeared lower compared to angiogenic OPCs, we still observed a marginally higher hypoxic response in *in vivo*

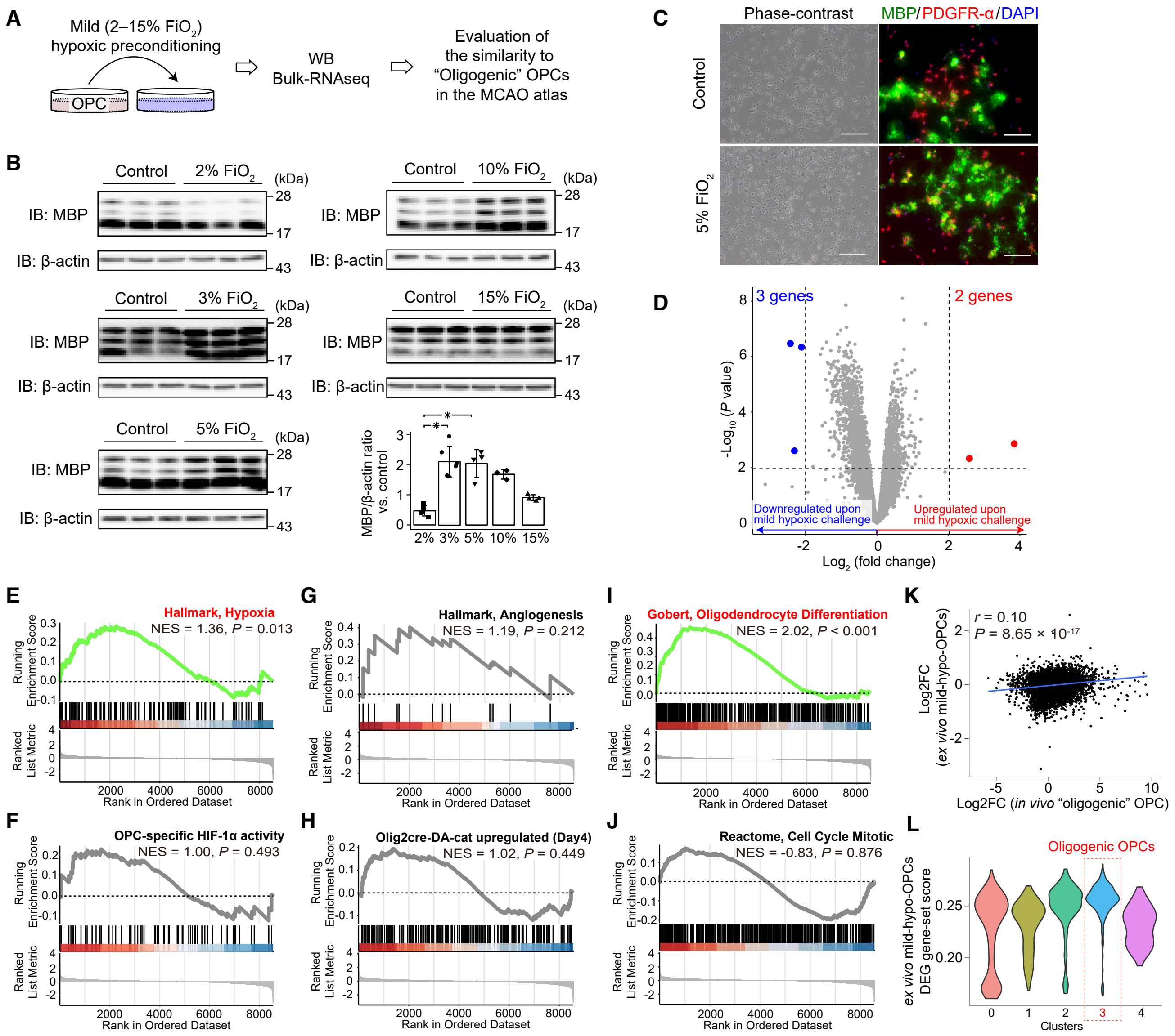

Figure 5. Mild hypoxic preconditioning directs *ex vivo* OPCs toward oligogenic OPCs

To examine how mild hypoxia affects OPC maturation, we conducted MBP western blotting (WB), immunostaining, and bulk RNA-seq, comparing *ex vivo* mild hypoxia-preconditioned OPCs (mild-hypo-OPCs) with *in vivo* oligogenic OPCs.

(A) Experimental design overview.

(B) WB analysis showed that FiO_2 levels of 3%–5% significantly increased MBP expression. Error bars represent standard deviation. Mann-Whitney U test with multiple comparisons adjusted using the Benjamini and Hochberg method, $p < 0.05$; $n = 5$ (control in 2%, biological replicates), $n = 5$ (2%, biological replicates), $n = 6$ (control in 3%, biological replicates), $n = 6$ (3%, biological replicates), $n = 4$ (control in 5%, biological replicates), $n = 4$ (5%, biological replicates), $n = 3$ (control in 10%, biological replicates), $n = 3$ (10%, biological replicates), $n = 4$ (control in 15%, biological replicates), $n = 4$ (15%, biological replicates).

(C) Immunostaining confirmed robust MBP induction at 5% FiO_2.

(D) Bulk RNA-seq-based differentially expressed gene (DEG) analysis revealed minimal transcriptomic changes in *ex vivo* mild-hypo-OPCs. $n = 3$ (per group, biological replicates).

(E–J) Gene set enrichment analysis (GSEA) showed increased activity in general hypoxia-related pathways (E), but no upregulation of HIF-1α signaling (F), angiogenesis (G), or WNT signaling (H). Importantly, GSEA revealed enhanced OPC differentiation toward oligodendrocytes (I), consistent with an oligogenic phenotype, without increases in mitotic activity (J).

(legend continued on next page)

oligogenic OPCs (cluster 3), as evidenced by OPC-specific HIF-1α activity (Figure 2A). This prompted us to investigate whether varying oxygen levels might differentially regulate OPC phenotypes and specifically contribute to the induction of *in vivo* oligogenic OPCs. To test this, we adjusted the oxygen concentration (2%, 3%, 5%, 10%, and 15% FiO_2) in our *ex vivo* OPC cultures and quantified MBP protein levels as a marker of OPC maturation (Figure 5A). Strikingly, MBP protein levels varied significantly with oxygen concentration, with 3%–5% oxygen most effectively increasing MBP levels (Figures 5B and 5C), indicating that mild hypoxic preconditioning may enhance OPC maturation *ex vivo*.

To confirm whether mild hypoxic preconditioning truly accelerates OPC maturation and to determine whether the transcriptomic changes resemble those of *in vivo* oligogenic OPCs, we conducted bulk RNA-seq analysis of these mild hypoxic-preconditioned OPCs (mild-hypo-OPCs). DEG analysis revealed minimal overall gene expression changes compared to those induced by severe hypoxic preconditioning (Figure 5D). GSEA and DEG analyses relative to standard OPCs showed that mild hypoxic preconditioning induced subtle hypoxia-associated transcriptomic changes (Figure 5E) but did not significantly increase HIF-1α activity (Figure 5F), angiogenesis (Figure 5G), or WNT pathway activity (Figure 5H). Consistent with the observed increase in MBP protein levels (Figure 5B), GSEA analysis demonstrated a clear promotion of OPC differentiation toward oligodendrocytes (Figure 5I). Although no upregulation of cell cycle activity was observed in mild-hypo-OPCs (Figure 5J), correlation analysis of log2 fold changes in DEGs between mild-hypo-OPCs and *in vivo* oligogenic OPCs revealed a weak but significant positive correlation (*rho* = 0.10, Figure 5K). Furthermore, gene set scoring analysis showed a mild upregulation of DEG scores from *ex vivo* mild-hypo-OPCs in *in vivo* oligogenic OPCs (Figure 5L).

Consistent with our prediction from the tMCAO atlas that the characteristics of *in vivo* oligogenic OPCs may be influenced by cytokines (Figure 2A), correlation analysis of log2 fold changes in DEGs between *ex vivo* IFN-γ-treated OPCs, DEX-treated OPCs, and *in vivo* oligogenic OPCs revealed significant positive correlations for IFN-γ treatment and negative correlations for DEX treatment (Figure S5A). Cell-cell interaction analysis of the tMCAO atlas indicated increased interaction strength between oligogenic OPCs and various glial cells, including microglia, astrocytes, and neurons (Figure S5B), consistent with the notion that oligogenic OPCs may promote myelination through interactions with multiple cell types during the subacute and chronic phases (Shi et al., 2021a). Thus, these findings collectively suggest that while mild hypoxia likely contributes, at least partially, to the emergence of *in vivo* oligogenic OPCs in the chronic stage post tMCAO, mild hypoxia preconditioning alone was insufficient to faithfully recapitulate the characteristics of *in vivo* oligogenic OPCs in our *ex vivo* OPC cultures. Therefore, cytokines and interactions with other cell types also play a crucial role in the induction of *in vivo* oligogenic OPCs.

DISCUSSION

In this study, by assembling and analyzing scRNA-seq data after tMCAO, we successfully transcriptionally characterized distinct OPC populations that preferentially emerged at specific time points following acute ischemic stroke. One population, termed angiogenic OPCs, appeared 3 days post tMCAO and was predicted to be induced by severe hypoxia. These OPCs appeared to be regulated by HIF-1α, promoting angiogenesis through the WNT and VEGF signaling pathways while also being attracted to endothelial cells via the CXCR4-CXCL12 axis. Notably, severe hypoxic preconditioning enabled *ex vivo* OPCs to acquire transcriptomes faithfully mirroring those of *in vivo* angiogenic OPCs. Importantly, transplantation of these *ex vivo* severe-hypo-OPCs effectively mitigated tMCAO-induced motor deficits and reduced infarct size, primarily through their pro-angiogenic activity. It is noteworthy that in the tMCAO model, at day 3–5 after stroke, the infarct is not yet fully matured; additional neuronal loss continues beyond this time point (Bellut et al., 2023; Buscemi et al., 2019). Therefore, it is reasonable to consider that OPC transplantation at day 3 may not directly rescue pan-necrotic tissue but could instead promote the initiation of repair processes, such as angiogenesis, thereby preventing ongoing secondary neuronal injury and facilitating tissue recovery. Another population, termed oligogenic OPCs, emerged 14 days post tMCAO and was predicted to contribute to myelination. Integration with *ex vivo* experiments suggested that mild hypoxic preconditioning partially contributed to the generation of *in vivo* oligogenic OPCs. Thus, our study highlights oxygen tone as a crucial determinant in OPCs acquiring protective phenotypes appropriate to specific time points after ischemic stroke, underscoring that a deep understanding and replication of this adaptive response are key to successfully developing OPC-based cell transplantation therapies.

(K) DEG $\log_2$ fold changes between *in vivo* oligogenic OPCs and *ex vivo* mild-hypo-OPCs were weakly but significantly correlated.
(L) Gene set scoring analysis in the transient middle cerebral artery occlusion (tMCAO) atlas showed a marginally increased DEG score of *ex vivo* mild-hypo-OPCs in oligogenic OPCs. NES, normalized enrichment score; Hallmark, Hallmark gene sets; DA-cat, dominant-active β-catenin; Reactome, Reactome pathway database.

Extensive previous research has documented the dynamic and complex biological changes occurring over the course of acute ischemic stroke, using various models, including the widely used tMCAO model. In the acute phase (within 24 h), there is a marked upregulation of inflammation- and apoptosis-related genes in the ischemic brain (Lu et al., 2004), driven by the infiltration of inflammatory cells, predominantly neutrophils and other innate immune cells (Cai et al., 2020), along with substantial neuronal loss (Buscemi et al., 2019; Garcia et al., 1995). In the subacute phase (3–7 days later), angiogenesis becomes detectable (Kanazawa et al., 2019). In the chronic phase, the infiltration of adaptive immune cells increases, and brain repair processes, such as remyelination, are activated (Ito et al., 2019; Shi et al., 2021a). Regarding OPCs, existing evidence indicates that OPCs proliferate, migrate toward the lesion, and accumulate in the peri-infarct region following cerebral ischemia (Bonfanti et al., 2017; Jiang et al., 2011). Notably, prior reports suggest that nearly all OPCs migrating into the peri-infarct region fail to differentiate or mature until the chronic phase (Bonfanti et al., 2017; Jiang et al., 2011), implying functional roles beyond myelination during the acute and subacute phases.

Despite this, surprisingly few studies have specifically examined the spatiotemporal transcriptional changes in OPCs after tMCAO. This lack of research likely stems from the fact that OPCs represent a relatively small and underrepresented cell population, making them challenging to study in isolation. Consequently, a meta-analysis approach, such as ours, provides a powerful strategy to uncover the dynamic changes within these rare cell populations (Oki et al., 2018; Zou et al., 2024). Importantly, our meta-analysis approach, for the first time at the transcriptomic level, highlights the remarkable adaptability of OPCs as they dynamically shift their functions—playing a critical role in angiogenesis during the subacute phase and contributing to remyelination during the chronic phase. Moreover, our study reveals that oxygen tone serves as a crucial factor sensed by OPCs to spatiotemporally acquire specific characteristics post stroke, a phenomenon partially shared with the developing brain (Allan et al., 2021; Yuen et al., 2014), to facilitate recovery after acute ischemic stroke. Critically, our tMCAO atlas revealed that these functional adaptations naturally occur in only a small subset of OPCs in the adult brain following ischemic stroke. This limited natural adaptation underscores the therapeutic potential of our proposed strategy—preconditioning OPCs *ex vivo* to induce "protective functional adaptations" before transplantation—positioning them as an ideal target for cell-based therapies aimed at enhancing stroke recovery. In this context, given that (1) both human and mouse studies have shown that post-stroke angiogenesis correlates with improved outcomes (Berkhemer et al., 2016; Greenberg, 2015), (2) our findings indicate that angiogenic OPCs are among the primary contributors to angiogenesis within brain-resident cells, and (3) severe hypoxic preconditioning *ex vivo* faithfully induced angiogenic OPCs, we propose that enhancing angiogenesis through the transplantation of *ex vivo* severely hypoxia-preconditioned angiogenic OPCs represents a theoretically robust strategy.

We have to acknowledge that although this study successfully characterized dynamic OPC functional adaptations at the transcriptomic level, many questions remain unanswered. The factors limiting the broader induction of angiogenic and oligogenic OPC phenotypes remain unclear. One possible contributor is inflammation, though other mechanisms may also play a role, warranting further investigation. Additionally, exploring the potential mutual transformation between angiogenic OPCs and oligogenic OPCs is particularly intriguing. Our results showed that transplanted angiogenic OPCs did not express maturation markers, even after entering the chronic phase post tMCAO, and trajectory analysis predicted distinct developmental paths for angiogenic and oligogenic OPCs.

Another unresolved question concerns the origin of angiogenic and oligogenic OPCs. Resident OPCs expand in the peri-infarct region, while subventricular zone (SVZ) progenitors can migrate into ischemic areas and generate oligodendrocyte lineage cells (Menn et al., 2006). Their relative contribution appears to be lesion dependent—peri-striatal infarcts involving more SVZ input—whereas distal cortical lesions may rely predominantly on resident OPCs, possibly with additional input from meningeal-derived OPCs (Maki et al., 2013). Our histochemical analyses showed that angiogenic OPCs preferentially reside in the peri-infarct cortex, whereas oligogenic OPCs were enriched at the peri-infarct corpus callosum with its adjacent regions, in proximity to the SVZ. These findings suggest that the origins of the two OPC subtypes may differ; however, rigorous lineage-tracing studies will be required to definitively resolve this question. Finally, we should acknowledge that although our study focused exclusively on tMCAO, other models—such as permanent middle cerebral artery occlusion (MCAO), thromboembolic occlusion, or photothrombosis—offer complementary strengths (Macrae, 2011; Matur et al., 2023), and future studies using these approaches will be valuable to confirm the external validity of our findings.

In conclusion, by assembling and analyzing scRNA-seq data, we delineated the temporal transcriptional changes of OPCs following ischemic stroke, identifying two functionally distinct populations: angiogenic OPCs, which support angiogenesis in the subacute phase, and oligogenic OPCs, which contribute to remyelination in the chronic phase. Furthermore, we successfully induced *ex vivo* OPCs with transcriptomic characteristics similar to angiogenic

OPCs through severe hypoxic preconditioning. Importantly, intravenous transplantation of these hypoxia-conditioned OPCs significantly enhanced post-stroke recovery by promoting angiogenesis in a stroke mouse model. Finally, we demonstrated that mild hypoxia partially contributes to the generation of oligogenic OPCs. Thus, after acute ischemic stroke, OPCs sense oxygen tone and undergo phenotypic shifts that enable them to fulfill stage-specific reparative roles, thereby facilitating recovery. Understanding and harnessing these adaptations offer promising therapeutic avenues for ischemic stroke, beyond current treatments that primarily focus on rapid reperfusion.

METHODS

Experimental design

We assembled publicly available scRNA-seq data from the *in vivo* tMCAO murine model to characterize OPC temporal transcriptomic change. Controlled laboratory experiments were then conducted, including immunohistochemical staining of the *in vivo* tMCAO murine model, *ex vivo* perturbations of OPCs, and their subsequent transplantation back into the *in vivo* tMCAO murine model.

tMCAO atlas

To identify relevant datasets, we searched the NCBI BioProject database on December 1, 2023, using the following search terms:

("tMCAO" [All Fields] AND "single cell" [All Fields]) OR ("middle cerebral artery occlusion" [All Fields] AND "single cell" [All Fields]) OR ("tMCAO" [All Fields] AND "scRNA-seq" [All Fields]) OR ("middle cerebral artery occlusion" [All Fields] AND "scRNAseq" [All Fields]).

Inclusion criteria required raw FASTQ data generated using the 10× Genomics Chromium platform to ensure uniform pipeline processing. Only datasets with multiple samples from multiple studies at the same time point were included to distinguish biological changes from batch effects. For eligible datasets, raw FASTQ files were mapped to the mouse reference transcriptome (mm10) using the pre-built reference from the 10× Genomics website (https://www.10xgenomics.com/) via the cellranger count command in CellRanger v.7.2.0. Each count matrix was analyzed using Seurat (v.5.0.1) in R (v.4.3.0), following best practices for scRNA-seq (Heumos et al., 2023). Detailed uniform quality control procedures and bioinformatics analyses for scRNA-seq data, as well as bulk RNA-seq data processing and analysis, are described in the supplemental methods.

Animals

Male C.B-17/Icr-+/+Jcl mice (10–12 weeks old, 18–33 g; Clea Japan, Tokyo, Japan, RRID: IMSR_JCL:JCL:mID-0004) were used. Our study exclusively examined male mice. It is unknown whether the findings are relevant for female mice.

tMCAO procedure

The standard intraluminal tMCAO method was employed (Kishida et al., 2019). Mice were anesthetized with 2%–4% isoflurane (FUJIFILM Wako, Osaka, Japan; catalog no. 099-06571) in a 50% N_2O/50% O_2 mixture. Body temperature was maintained at 37°C–38°C using a heating pad and lamp (UNIQUE MEDICAL, Tokyo, Japan; catalog no. ATC-101B-MS). A midline incision was made to expose the left common carotid artery, where a 0.22 mm Doccol filament (Doccol, Sharon, MA, USA; catalog no. 602256PK10) was inserted up to the middle cerebral artery origin. Reperfusion was established after 60 min by filament withdrawal. tMCAO success was confirmed by laser Doppler flowmetry (Omegawave, Tokyo, Japan; catalog no. FLO-C1), ensuring at least an 80% reduction in cerebral blood flow during ischemia. Of 28 mice, 20 underwent successful tMCAO, while 8 died postoperatively.

Immunohistochemistry

Mouse brains were collected after perfusion with PBS (Nacalai Tesque, Kyoto, Japan; catalog no. 14249-24) and 4% PFA (Nacalai Tesque; catalog no. 09154-85) and then fixed in 4% PFA for 24 h and preserved in 20% sucrose. Coronal sections (20 μm) were rinsed with PBS, blocked with 3% BSA (Nacalai Tesque; catalog no. 01281-26), and incubated overnight at 4°C in 0.3% BSA with primary antibodies, including the following:

(1) Anti-MAP-2 (Proteintech; catalog no. 17490-1-AP; RRID: AB_2137880) at 1:500
(2) Anti-PDGFRα (Bio-Techne; catalog no. AF1062; RRID: AB_2236897) at 1:200
(3) Anti-glucose transporter-1 (GLUT-1) (Merck Millipore; catalog no. 07–1401; RRID: AB_1587074) at 1:200
(4) Anti-GST-π (MBL; catalog no. 311-H; RRID: AB_591790) at 1:200
(5) Anti-doublecortin (Abcam; catalog no. ab18723; RRID: AB_732011) at 1:200
(6) Anti-Iba-1 (FUJIFILM Wako; catalog no. 011-27991; RRID: AB_2935833) at 1:200

After three PBS washes, sections were incubated for 1 hour at room temperature with secondary antibodies, including the following:

(1) Anti-rabbit Alexa Fluor 594 (Thermo Fisher Scientific; catalog no. A-21207; RRID: AB_141637) at 1:500
(2) Anti-goat Alexa Fluor 594 (Thermo Fisher Scientific; catalog no. A-11058; RRID: AB_2534105) at 1:500

The sections were washed again, mounted with DAPI (Thermo Fisher Scientific; catalog no. 62247), and imaged using a fluorescence microscope (KEYENCE BZ-X710, Keyence, Osaka, Japan) or a confocal microscope (Olympus FV1000, Olympus, Tokyo, Japan).

Isolation of primary oligodendrocyte lineage cells

OPCs were isolated as previously described (Kishida et al., 2019). Cerebral cortices from P1 or P2 wild-type Sprague-Dawley rats (Shimizu Laboratory Supplies, Kyoto, Japan; RRID:MGI:5651135) were dissected, minced, and digested into a single-cell suspension using 0.25% trypsin-EDTA (Thermo Fisher Scientific; catalog no.25200056) and DNase I (Sigma-Aldrich; catalog no. D5219-500μg) at 37°C for 15 min. After filtration through a 40-μm cell strainer (Corning; catalog no. 352340), cells were plated in poly-D-lysine-coated (Sigma-Aldrich; catalog no. P0421-100MG) flasks with DMEM (FUJIFILM Wako; catalog no. 043-30085), 1% penicillin/streptomycin (Nacalai Tesque; catalog no. 26253-84), and 20% heat-inactivated fetal bovine serum (Biosera; catalog no. FB-1365/500). Once confluent (∼10 days), microglia were removed by shaking at 220 rpm for 1 h at 37°C, followed by a medium change and overnight shaking (20 h). Non-adherent cells were plated on uncoated culture dishes for 1 h at 37°C to eliminate residual astrocytes and microglia. The remaining cells were seeded on poly-L-ornithine-coated (Sigma-Aldrich; catalog no. P3655-100MG) plates at 20,000 cells/cm^2 in Neurobasal Medium (Thermo Fisher Scientific; catalog no. 21103049) with 1% penicillin/streptomycin, 2 mM glutamine (Nacalai Tesque; catalog no.16948-04), 10 ng/mL FGF-2 (Thermo Fisher Scientific; catalog no. PTI-100-18B-50), 10 ng/mL PDGF-AA (Thermo Fisher Scientific; catalog no.PTI-100-13A-10), and 2% B27 supplement (Thermo Fisher Scientific; catalog no.17504044). Six days post plating, OPCs underwent 0.1% oxygen-glucose deprivation for 6 h to induce severe hypoxia, followed by exposure to 2%, 3%, 5%, 10%, or 15% FiO_2 for 6 days under mild hypoxia. Due to the high cytotoxicity of 0.1% oxygen-glucose deprivation, cells were only assessed at 6 h. For transplantation, hypoxic-preconditioned OPCs were dissociated using 5 mL Accumax (Innovative Cell Technologies; catalog no. AM105-500ML), and 7.5×10^5 cells were suspended in 5 μL PBS.

OPC transplantation

tMCAO was performed in 32 mice, with 8 excluded due to death before day 3. The remaining 24 mice were randomly assigned on day 3 to one of three groups: control ($n = 12$), standard OPC ($n = 6$), and severe-hypo-OPC ($n = 6$). Under anesthesia, all mice received orbital injections as follows:

(1) Control group: 5 μL PBS
(2) Standard OPC group: 7.5×10^5 CAG-EGFP-tagged standard OPCs in 5 μL PBS
(3) Severe-hypo-OPC group: 7.5×10^5 CAG-EGFP-tagged severe-hypo-OPCs in 5 μL PBS

All mice received intraperitoneal FK506 (1 mg/kg/day; FUJIFILM Wako; catalog no. 063-06071) and 500 μL normal saline daily from days 1–7 post tMCAO.

Neurobehavioral and infarct volume measurements

We assessed mNSS by a blinded evaluator on days 3, 7, 10, and 14, following a standardized 0–18 scale that includes motor, sensory, beam balance, and reflex tests. Rotarod tests were conducted on the same days using an accelerating rotarod (Ugo Basile; catalog no. 47650) set to increase from 0 to 40 rpm over 4 min. The average time to fall was recorded over two trials. Mice were sacrificed on day 14, and brains were sectioned into 20 μm coronal slices. Five sections (+1.0, +0.5, 0, −0.5, and −1.0 mm relative to bregma) were stained with anti-MAP-2. A blinded investigator captured images using a fluorescence microscope (KEYENCE BZ-X710) and analyzed them in TIFF format using ImageJ 1.53a. The infarct area was calculated as follows:

(1) The contralateral hemisphere area (A) and the ipsilateral hemisphere area excluding the infarct zone (B) were measured.
(2) Infarct area = (A − B)/A (%).
(3) Infarct areas were multiplied by the section interval (0.5 mm) and summed to determine total infarct volume.

Evaluation of angiogenesis and oligodendrogenesis

Coronal brain sections (20 μm) were stained with anti-GLUT-1 (1:200) or anti-GST-π (1:200). Four penumbra regions surrounding the infarct were analyzed in sections at +0.5, 0, and −0.5 mm relative to the bregma. Images were captured at 20× magnification and analyzed using ImageJ. Angiogenesis was assessed by counting the total number of vessel branches in 12 regions (4 per section, 3 sections). Oligodendrogenesis was quantified by counting GST-π-positive oligodendrocytes in the same regions.

Evaluation of spatial distribution of angiogenic and oligogenic OPCs

Coronal brain sections (20 μm) were stained with anti-BCAS1 (1:200), anti-VEGFA (1:200), and anti-OLIG2 (1:200). Four penumbra regions surrounding the infarct were analyzed in sections at +0.5 mm relative to the bregma. Images were captured at 20× magnification and analyzed. On day 3 after MCAO, the proportion of $VEGFA^+$ $OLIG2^+$ angiogenic OPCs among total $OLIG2^+$ oligodendrocyte lineage cells was compared between the

peri-infarct cortex and the peri-infarct corpus callosum with its adjacent regions. On day 14 after MCAO, the proportion of BCAS1$^+$ OLIG2$^+$ oligogenic OPCs was compared between these regions.

Statistical analysis

Statistical analyses were primarily conducted by K.T., who is certified by the Japan Statistical Society (grade 2), using custom R scripts. The Mann-Whitney U test and Spearman's rank correlation coefficients (*rho*) were used for between-group comparisons and correlation analyses, respectively, as the assumption of normal distribution was often not met. Throughout the study, multiple testing was adjusted using the Benjamini and Hochberg method. Two-sided p values <0.05 were considered statistically significant. Values are presented as mean ± standard deviation.

Study approval

All procedures adhered to Kyoto University's animal experimentation guidelines and were approved by its Ethical Committee.

RESOURCE AVAILABILITY

Lead contact

Further information and requests for resources and reagents should be directed to and will be fulfilled by the lead contact, Takakuni Maki (harutoma@kuhp.kyoto-u.ac.jp).

Materials availability

All materials and lines generated in this study are available from the lead contact.

Data and code availability

- All data are available in the main text or supplemental information including Supporting Data Values file. The MCAO atlas datasets—including both the full dataset and the OPC-only subset—have been deposited in the Single-Cell Portal under accession numbers SCP3078 (https://singlecell.broadinstitute.org/single_cell/study/SCP3078) and SCP3080 (https://singlecell.broadinstitute.org/single_cell/study/SCP3080), respectively.
- All R code, statistical source data, and data used to generate the figures have been deposited in the Open Science Framework (https://osf.io/a3tc8/).
- Sequence data generated in our study have been deposited in GEO under accession number GSE275670.

ACKNOWLEDGMENTS

The authors would like to thank other members of our department for their support. This work was funded by the Ministry of Education, Culture, Sports, Science and Technology Japan (Grant-in-Aid for Scientific Research C) 20K06853 (T.M.), Japan Science and Technology Agency (Moonshot R&D) JPMJMS2024 (T.M.), and Japan Society for the Promotion of Science (Grant-in-Aid for Young Scientists) JP22K18178 (K.T.). The graphical abstract preparation was supported by Editage.

AUTHOR CONTRIBUTIONS

Conceptualization, Y.K., K.Y., K.T., and T.M.; methodology, Y.K., K.Y., K.T., and T.M.; investigation, Y.K., K.Y., K.T., and T.M.; visualization, Y.K., K.T., and A.K.; funding acquisition, K.T. and T.M.; project administration, R.T. and T.M.; supervision, R.T. and T.M.; writing – original draft, Y.K., K.T., and T.M.; writing – review and editing, Y.K., K.T., and T.M.

DECLARATION OF INTERESTS

The authors declare no competing interests.

SUPPLEMENTAL INFORMATION

Supplemental information can be found online at https://doi.org/10.1016/j.stemcr.2025.102687.

Received: June 5, 2025
Revised: September 29, 2025
Accepted: September 30, 2025
Published: October 30, 2025

REFERENCES

Akay, L.A., Effenberger, A.H., and Tsai, L.-H. (2021). Cell of all trades: oligodendrocyte precursor cells in synaptic, vascular, and immune function. Genes Dev. *35*, 180–198. https://doi.org/10.1101/gad.344218.120.

Allan, K.C., Hu, L.R., Scavuzzo, M.A., Morton, A.R., Gevorgyan, A.S., Cohn, E.F., Clayton, B.L.L., Bederman, I.R., Hung, S., Bartels, C.F., et al. (2021). Non-canonical Targets of HIF1a Impair Oligodendrocyte Progenitor Cell Function. Cell Stem Cell *28*, 257–272.e11. https://doi.org/10.1016/j.stem.2020.09.019.

Anthony, S., Cabantan, D., Monsour, M., and Borlongan, C.V. (2022). Neuroinflammation, Stem Cells, and Stroke. Stroke *53*, 1460–1472. https://doi.org/10.1161/STROKEAHA.121.036948.

Baranova, O., Miranda, L.F., Pichiule, P., Dragatsis, I., Johnson, R.S., and Chavez, J.C. (2007). Neuron-specific inactivation of the hypoxia inducible factor 1 alpha increases brain injury in a mouse model of transient focal cerebral ischemia. J. Neurosci. *27*, 6320–6332. https://doi.org/10.1523/JNEUROSCI.0449-07.2007.

Bellut, M., Bieber, M., Kraft, P., Weber, A.N.R., Stoll, G., and Schuhmann, M.K. (2023). Delayed NLRP3 inflammasome inhibition ameliorates subacute stroke progression in mice. J. Neuroinflammation *20*, 4. https://doi.org/10.1186/s12974-022-02674-w.

Bergles, D.E., and Richardson, W.D. (2015). Oligodendrocyte Development and Plasticity. Cold Spring Harbor Perspect. Biol. *8*, a020453. https://doi.org/10.1101/cshperspect.a020453.

Berkhemer, O.A., Jansen, I.G.H., Beumer, D., Fransen, P.S.S., van den Berg, L.A., Yoo, A.J., Lingsma, H.F., Sprengers, M.E.S., Jenniskens, S.F.M., Lycklama À Nijeholt, G.J., et al. (2016). Collateral Status on Baseline Computed Tomographic Angiography and Intra-Arterial Treatment Effect in Patients With Proximal Anterior

Circulation Stroke. Stroke *47*, 768–776. https://doi.org/10.1161/STROKEAHA.115.011788.

Bondareva, O., Rodríguez-Aguilera, J.R., Oliveira, F., Liao, L., Rose, A., Gupta, A., Singh, K., Geier, F., Schuster, J., Boeckel, J.-N., et al. (2022). Single-cell profiling of vascular endothelial cells reveals progressive organ-specific vulnerabilities during obesity. Nat. Metab. *4*, 1591–1610. https://doi.org/10.1038/s42255-022-00674-x.

Bonfanti, E., Gelosa, P., Fumagalli, M., Dimou, L., Viganò, F., Tremoli, E., Cimino, M., Sironi, L., and Abbracchio, M.P. (2017). The role of oligodendrocyte precursor cells expressing the GPR17 receptor in brain remodeling after stroke. Cell Death Dis. *8*, e2871. https://doi.org/10.1038/cddis.2017.256.

Buscemi, L., Price, M., Bezzi, P., and Hirt, L. (2019). Spatio-temporal overview of neuroinflammation in an experimental mouse stroke model. Sci. Rep. *9*, 507. https://doi.org/10.1038/s41598-018-36598-4.

Cai, W., Liu, S., Hu, M., Huang, F., Zhu, Q., Qiu, W., Hu, X., Colello, J., Zheng, S.G., and Lu, Z. (2020). Functional Dynamics of Neutrophils After Ischemic Stroke. Transl. Stroke Res. *11*, 108–121. https://doi.org/10.1007/s12975-019-00694-y.

Castanza, A.S., Recla, J.M., Eby, D., Thorvaldsdóttir, H., Bult, C.J., and Mesirov, J.P. (2023). Extending support for mouse data in the Molecular Signatures Database (MSigDB). Nat. Methods *20*, 1619–1620. https://doi.org/10.1038/s41592-023-02014-7.

Chamorro, Á., Lo, E.H., Renú, A., van Leyen, K., and Lyden, P.D. (2021). The future of neuroprotection in stroke. J. Neurol. Neurosurg. Psychiatry *92*, 129–135. https://doi.org/10.1136/jnnp-2020-324283.

Chen, L.-X., Ma, S.-M., Zhang, P., Fan, Z.-C., Xiong, M., Cheng, G.-Q., Yang, Y., Qiu, Z.-L., Zhou, W.-H., and Li, J. (2015). Neuroprotective effects of oligodendrocyte progenitor cell transplantation in premature rat brain following hypoxic-ischemic injury. PLoS One *10*, e0115997. https://doi.org/10.1371/journal.pone.0115997.

Clarkson, A.N., Huang, B.S., Macisaac, S.E., Mody, I., and Carmichael, S.T. (2010). Reducing excessive GABA-mediated tonic inhibition promotes functional recovery after stroke. Nature *468*, 305–309. https://doi.org/10.1038/nature09511.

Doll, D.N., Barr, T.L., and Simpkins, J.W. (2014). Cytokines: their role in stroke and potential use as biomarkers and therapeutic targets. Aging Dis. *5*, 294–306. https://doi.org/10.14336/AD.2014.0500294.

Emery, B., Agalliu, D., Cahoy, J.D., Watkins, T.A., Dugas, J.C., Mulinyawe, S.B., Ibrahim, A., Ligon, K.L., Rowitch, D.H., and Barres, B.A. (2009). Myelin gene regulatory factor is a critical transcriptional regulator required for CNS myelination. Cell *138*, 172–185. https://doi.org/10.1016/j.cell.2009.04.031.

Fancy, S.P.J., Baranzini, S.E., Zhao, C., Yuk, D.-I., Irvine, K.-A., Kaing, S., Sanai, N., Franklin, R.J.M., and Rowitch, D.H. (2009). Dysregulation of the Wnt pathway inhibits timely myelination and remyelination in the mammalian CNS. Genes Dev. *23*, 1571–1585. https://doi.org/10.1101/gad.1806309.

Fancy, S.P.J., Harrington, E.P., Baranzini, S.E., Silbereis, J.C., Shiow, L.R., Yuen, T.J., Huang, E.J., Lomvardas, S., and Rowitch, D.H. (2014). Parallel states of pathological Wnt signaling in neonatal brain injury and colon cancer. Nat. Neurosci. *17*, 506–512. https://doi.org/10.1038/nn.3676.

Fang, J., Wang, Z., and Miao, C.-Y. (2023). Angiogenesis after ischemic stroke. Acta Pharmacol. Sin. *44*, 1305–1321. https://doi.org/10.1038/s41401-023-01061-2.

Fard, M.K., van der Meer, F., Sánchez, P., Cantuti-Castelvetri, L., Mandad, S., Jäkel, S., Fornasiero, E.F., Schmitt, S., Ehrlich, M., Starost, L., et al. (2017). BCAS1 expression defines a population of early myelinating oligodendrocytes in multiple sclerosis lesions. Sci. Transl. Med. *9*, eaam7816. https://doi.org/10.1126/scitranslmed.aam7816.

Frazier, A.P., Mitchell, D.N., Given, K.S., Hunn, G., Burch, A.M., Childs, C.R., Moreno-Garcia, M., Corigilano, M.R., Quillinan, N., Macklin, W.B., et al. (2023). Chronic changes in oligodendrocyte sub-populations after middle cerebral artery occlusion in neonatal mice. Glia *71*, 1429–1450. https://doi.org/10.1002/glia.24349.

Garcia, J.H., Liu, K.F., and Ho, K.L. (1995). Neuronal necrosis after middle cerebral artery occlusion in Wistar rats progresses at different time intervals in the caudoputamen and the cortex. Stroke *26*, 636–643. https://doi.org/10.1161/01.str.26.4.636.

Greenberg, D.A. (2015). Poststroke angiogenesis, pro: making the desert bloom. Stroke *46*, e101–e102. https://doi.org/10.1161/STROKEAHA.114.007641.

Hamanaka, G., Hernández, I.C., Takase, H., Ishikawa, H., Benboujja, F., Kimura, S., Fukuda, N., Guo, S., Lok, J., Lo, E.H., and Arai, K. (2023). Myelination- and migration-associated genes are downregulated after phagocytosis in cultured oligodendrocyte precursor cells. J. Neurochem. *167*, 571–581. https://doi.org/10.1111/jnc.15994.

Hase, Y., Ameen-Ali, K.E., Waller, R., Simpson, J.E., Stafford, C., Mahesh, A., Ryan, L., Pickering, L., Bodman, C., Hase, M., et al. (2022). Differential perivascular microglial activation in the deep white matter in vascular dementia developed post-stroke. Brain Pathol. *32*, e13101. https://doi.org/10.1111/bpa.13101.

He, Q., Ma, Y., Liu, J., Zhang, D., Ren, J., Zhao, R., Chang, J., Guo, Z.-N., and Yang, Y. (2021). Biological Functions and Regulatory Mechanisms of Hypoxia-Inducible Factor-1α in Ischemic Stroke. Front. Immunol. *12*, 801985. https://doi.org/10.3389/fimmu.2021.801985.

Heumos, L., Schaar, A.C., Lance, C., Litinetskaya, A., Drost, F., Zappia, L., Lücken, M.D., Strobl, D.C., Henao, J., Curion, F., et al. (2023). Best practices for single-cell analysis across modalities. Nat. Rev. Genet. *24*, 550–572. https://doi.org/10.1038/s41576-023-00586-w.

Hosoki, S., Saito, S., Tonomura, S., Ishiyama, H., Yoshimoto, T., Ikeda, S., Ikenouchi, H., Yamamoto, Y., Hattori, Y., Miwa, K., et al. (2020). Oral Carriage of Streptococcus mutans Harboring the cnm Gene Relates to an Increased Incidence of Cerebral Microbleeds. Stroke *51*, 3632–3639. https://doi.org/10.1161/STROKEAHA.120.029607.

Houkin, K., Osanai, T., Uchiyama, S., Minematsu, K., Taguchi, A., Maruichi, K., Niiya, Y., Asaoka, K., Kuga, Y., Takizawa, K., et al. (2024). Allogeneic Stem Cell Therapy for Acute Ischemic Stroke: The Phase 2/3 TREASURE Randomized Clinical Trial. JAMA Neurol. *81*, 154–162. https://doi.org/10.1001/jamaneurol.2023.5200.

Hu, B., Pei, J., Wan, C., Liu, S., Xu, Z., Zou, Y., Li, Z., and Tang, Z. (2024). Mechanisms of Postischemic Stroke Angiogenesis: A Multifaceted Approach. J. Inflamm. Res. *17*, 4625–4646. https://doi.org/10.2147/JIR.S461427.

Ito, M., Komai, K., Mise-Omata, S., Iizuka-Koga, M., Noguchi, Y., Kondo, T., Sakai, R., Matsuo, K., Nakayama, T., Yoshie, O., et al. (2019). Brain regulatory T cells suppress astrogliosis and potentiate neurological recovery. Nature *565*, 246–250. https://doi.org/10.1038/s41586-018-0824-5.

Jiang, L., Shen, F., Degos, V., Schonemann, M., Pleasure, S.J., Mellon, S.H., Young, W.L., and Su, H. (2011). Oligogenesis and oligodendrocyte progenitor maturation vary in different brain regions and partially correlate with local angiogenesis after ischemic stroke. Transl. Stroke Res. *2*, 366–375. https://doi.org/10.1007/s12975-011-0078-0.

Kakae, M., Nakajima, H., Tobori, S., Kawashita, A., Miyanohara, J., Morishima, M., Nagayasu, K., Nakagawa, T., Shigetomi, E., Koizumi, S., et al. (2023). The astrocytic TRPA1 channel mediates an intrinsic protective response to vascular cognitive impairment via LIF production. Sci. Adv. *9*, eadh0102. https://doi.org/10.1126/sciadv.adh0102.

Kanazawa, M., Takahashi, T., Ishikawa, M., Onodera, O., Shimohata, T., and Del Zoppo, G.J. (2019). Angiogenesis in the ischemic core: A potential treatment target? J. Cerebr. Blood Flow Metabol. *39*, 753–769. https://doi.org/10.1177/0271678X19834158.

Kim, S., Lee, W., Jo, H., Sonn, S.-K., Jeong, S.-J., Seo, S., Suh, J., Jin, J., Kweon, H.Y., Kim, T.K., et al. (2022). The antioxidant enzyme Peroxiredoxin-1 controls stroke-associated microglia against acute ischemic stroke. Redox Biol. *54*, 102347. https://doi.org/10.1016/j.redox.2022.102347.

Kishida, N., Maki, T., Takagi, Y., Yasuda, K., Kinoshita, H., Ayaki, T., Noro, T., Kinoshita, Y., Ono, Y., Kataoka, H., et al. (2019). Role of Perivascular Oligodendrocyte Precursor Cells in Angiogenesis After Brain Ischemia. J. Am. Heart Assoc. *8*, e011824. https://doi.org/10.1161/JAHA.118.011824.

Krupinski, J., Kaluza, J., Kumar, P., Kumar, S., and Wang, J.M. (1994). Role of angiogenesis in patients with cerebral ischemic stroke. Stroke *25*, 1794–1798. https://doi.org/10.1161/01.str.25.9.1794.

Kumar Podder, A., Mohamed, M.A., Seidman, R.A., Tseropoulos, G., Polanco, J.J., Lei, P., Sim, F.J., and Andreadis, S.T. (2024). Injectable shear-thinning hydrogels promote oligodendrocyte progenitor cell survival and remyelination in the central nervous system. Sci. Adv. *10*, eadk9918. https://doi.org/10.1126/sciadv.adk9918.

Li, H.L., Kostulas, N., Huang, Y.M., Xiao, B.G., van der Meide, P., Kostulas, V., Giedraitas, V., and Link, H. (2001). IL-17 and IFN-gamma mRNA expression is increased in the brain and systemically after permanent middle cerebral artery occlusion in the rat. J. Neuroimmunol. *116*, 5–14. https://doi.org/10.1016/s0165-5728(01)00264-8.

Li, W., He, T., Shi, R., Song, Y., Wang, L., Zhang, Z., Tang, Y., Yang, G.-Y., and Wang, Y. (2021). Oligodendrocyte Precursor Cells Transplantation Improves Stroke Recovery via Oligodendrogenesis, Neurite Growth and Synaptogenesis. Aging Dis. *12*, 2096–2112. https://doi.org/10.14336/AD.2021.0416.

Liu, Y., Huang, J., Pandey, R., Liu, P., Therani, B., Qiu, Q., Rao, S., Geurts, A.M., Cowley, A.W., Greene, A.S., and Liang, M. (2023). Robustness of single-cell RNA-seq for identifying differentially expressed genes. BMC Genom. *24*, 371. https://doi.org/10.1186/s12864-023-09487-y.

Lu, X.-C.M., Williams, A.J., Yao, C., Berti, R., Hartings, J.A., Whipple, R., Vahey, M.T., Polavarapu, R.G., Woller, K.L., Tortella, F.C., and Dave, J.R. (2004). Microarray analysis of acute and delayed gene expression profile in rats after focal ischemic brain injury and reperfusion. J. Neurosci. Res. *77*, 843–857. https://doi.org/10.1002/jnr.20218.

Macrae, I.M. (2011). Preclinical stroke research–advantages and disadvantages of the most common rodent models of focal ischaemia. Br. J. Pharmacol. *164*, 1062–1078. https://doi.org/10.1111/j.1476-5381.2011.01398.x.

Maki, T., Liang, A.C., Miyamoto, N., Lo, E.H., and Arai, K. (2013). Mechanisms of oligodendrocyte regeneration from ventricular-subventricular zone-derived progenitor cells in white matter diseases. Front. Cell. Neurosci. *7*, 275. https://doi.org/10.3389/fncel.2013.00275.

Marques, S., Zeisel, A., Codeluppi, S., van Bruggen, D., Mendanha Falcão, A., Xiao, L., Li, H., Häring, M., Hochgerner, H., Romanov, R.A., et al. (2016). Oligodendrocyte heterogeneity in the mouse juvenile and adult central nervous system. Science *352*, 1326–1329. https://doi.org/10.1126/science.aaf6463.

Matur, A.V., Candelario-Jalil, E., Paul, S., Karamyan, V.T., Lee, J.D., Pennypacker, K., and Fraser, J.F. (2023). Translating Animal Models of Ischemic Stroke to the Human Condition. Transl. Stroke Res. *14*, 842–853. https://doi.org/10.1007/s12975-022-01082-9.

Meijer, M., Agirre, E., Kabbe, M., van Tuijn, C.A., Heskol, A., Zheng, C., Mendanha Falcão, A., Bartosovic, M., Kirby, L., Calini, D., et al. (2022). Epigenomic priming of immune genes implicates oligodendroglia in multiple sclerosis susceptibility. Neuron *110*, 1193–1210.e13. https://doi.org/10.1016/j.neuron.2021.12.034.

Menn, B., Garcia-Verdugo, J.M., Yaschine, C., Gonzalez-Perez, O., Rowitch, D., and Alvarez-Buylla, A. (2006). Origin of oligodendrocytes in the subventricular zone of the adult brain. J. Neurosci. *26*, 7907–7918. https://doi.org/10.1523/JNEUROSCI.1299-06.2006.

Minocha, S., Valloton, D., Brunet, I., Eichmann, A., Hornung, J.-P., and Lebrand, C. (2015). NG2 glia are required for vessel network formation during embryonic development. eLife *4*, e09102. https://doi.org/10.7554/eLife.09102.

Müller-Dott, S., Tsirvouli, E., Vazquez, M., Ramirez Flores, R.O., Badia-I-Mompel, P., Fallegger, R., Türei, D., Lægreid, A., and Saez-Rodriguez, J. (2023). Expanding the coverage of regulons from high-confidence prior knowledge for accurate estimation of transcription factor activities. Nucleic Acids Res. *51*, 10934–10949. https://doi.org/10.1093/nar/gkad841.

Nakahashi-Oda, C., Fujiyama, S., Nakazawa, Y., Kanemaru, K., Wang, Y., Lyu, W., Shichita, T., Kitaura, J., Abe, F., and Shibuya, A. (2021). CD300a blockade enhances efferocytosis by infiltrating myeloid cells and ameliorates neuronal deficit after ischemic stroke. Sci. Immunol. *6*, eabe7915. https://doi.org/10.1126/sciimmunol.abe7915.

Okazaki, S., Morimoto, T., Kamatani, Y., Kamimura, T., Kobayashi, H., Harada, K., Tomita, T., Higashiyama, A., Takahashi, J.C., Nakagawara, J., et al. (2019). Moyamoya Disease Susceptibility Variant RNF213 p.R4810K Increases the Risk of Ischemic Stroke Attributable to Large-Artery Atherosclerosis. Circulation *139*, 295–298. https://doi.org/10.1161/CIRCULATIONAHA.118.038439.

Oki, S., Ohta, T., Shioi, G., Hatanaka, H., Ogasawara, O., Okuda, Y., Kawaji, H., Nakaki, R., Sese, J., and Meno, C. (2018). ChIP-Atlas: a data-mining suite powered by full integration of public ChIP-seq data. EMBO Rep. *19*, e46255. https://doi.org/10.15252/embr.201846255.

Qian, Z., Li, H., Yang, H., Yang, Q., Lu, Z., Wang, L., Chen, Y., and Li, X. (2021). Osteocalcin attenuates oligodendrocyte differentiation and myelination via GPR37 signaling in the mouse brain. Sci. Adv. *7*, eabi5811. https://doi.org/10.1126/sciadv.abi5811.

Rybnikova, E.A., Nalivaeva, N.N., Zenko, M.Y., and Baranova, K.A. (2022). Intermittent Hypoxic Training as an Effective Tool for Increasing the Adaptive Potential, Endurance and Working Capacity of the Brain. Front. Neurosci. *16*, 941740. https://doi.org/10.3389/fnins.2022.941740.

Shi, L., Sun, Z., Su, W., Xu, F., Xie, D., Zhang, Q., Dai, X., Iyer, K., Hitchens, T.K., Foley, L.M., et al. (2021a). Treg cell-derived osteopontin promotes microglia-mediated white matter repair after ischemic stroke. Immunity *54*, 1527–1542.e8. https://doi.org/10.1016/j.immuni.2021.04.022.

Shi, X., Luo, L., Wang, J., Shen, H., Li, Y., Mamtilahun, M., Liu, C., Shi, R., Lee, J.-H., Tian, H., et al. (2021b). Stroke subtype-dependent synapse elimination by reactive gliosis in mice. Nat. Commun. *12*, 6943. https://doi.org/10.1038/s41467-021-27248-x.

Tiedt, S., Buchan, A.M., Dichgans, M., Lizasoain, I., Moro, M.A., and Lo, E.H. (2022). The neurovascular unit and systemic biology in stroke - implications for translation and treatment. Nat. Rev. Neurol. *18*, 597–612. https://doi.org/10.1038/s41582-022-00703-z.

Tsai, H.-H., Niu, J., Munji, R., Davalos, D., Chang, J., Zhang, H., Tien, A.-C., Kuo, C.J., Chan, J.R., Daneman, R., and Fancy, S.P.J. (2016). Oligodendrocyte precursors migrate along vasculature in the developing nervous system. Science *351*, 379–384. https://doi.org/10.1126/science.aad3839.

Wang, L.-P., Pan, J., Li, Y., Geng, J., Liu, C., Zhang, L.-Y., Zhou, P., Tang, Y.-H., Wang, Y., Zhang, Z., and Yang, G.Y. (2022). Oligodendrocyte precursor cell transplantation promotes angiogenesis and remyelination via Wnt/β-catenin pathway in a mouse model of middle cerebral artery occlusion. J. Cerebr. Blood Flow Metabol. *42*, 757–770. https://doi.org/10.1177/0271678X211065391.

Wu, D.-M., Liu, J.-P., Liu, J., Ge, W.-H., Wu, S.-Z., Zeng, C.-J., Liang, J., Liu, K., Lin, Q., Hong, X.-W., et al. (2023). Immune pathway activation in neurons triggers neural damage after stroke. Cell Rep. *42*, 113368. https://doi.org/10.1016/j.celrep.2023.113368.

Xiao, Y., and Czopka, T. (2023). Myelination-independent functions of oligodendrocyte precursor cells in health and disease. Nat. Neurosci. *26*, 1663–1669. https://doi.org/10.1038/s41593-023-01423-3.

Yuen, T.J., Silbereis, J.C., Griveau, A., Chang, S.M., Daneman, R., Fancy, S.P.J., Zahed, H., Maltepe, E., and Rowitch, D.H. (2014). Oligodendrocyte-encoded HIF function couples postnatal myelination and white matter angiogenesis. Cell *158*, 383–396. https://doi.org/10.1016/j.cell.2014.04.052.

Zeng, F., Cao, J., Hong, Z., Liu, Y., Hao, J., Qin, Z., Zou, X., and Tao, T. (2023). Single-cell analyses reveal the dynamic functions of Itgb2+ microglia subclusters at different stages of cerebral ischemia-reperfusion injury in transient middle cerebral occlusion mice model. Front. Immunol. *14*, 1114663. https://doi.org/10.3389/fimmu.2023.1114663.

Zhang, K., Zhu, L., and Fan, M. (2011). Oxygen, a Key Factor Regulating Cell Behavior during Neurogenesis and Cerebral Diseases. Front. Mol. Neurosci. *4*, 5. https://doi.org/10.3389/fnmol.2011.00005.

Zheng, K., Lin, L., Jiang, W., Chen, L., Zhang, X., Zhang, Q., Ren, Y., and Hao, J. (2022). Single-cell RNA-seq reveals the transcriptional landscape in ischemic stroke. J. Cerebr. Blood Flow Metabol. *42*, 56–73. https://doi.org/10.1177/0271678X211026770.

Zou, Z., Ohta, T., and Oki, S. (2024). ChIP-Atlas 3.0: a data-mining suite to explore chromosome architecture together with large-scale regulome data. Nucleic Acids Res. *52*, W45–W53. https://doi.org/10.1093/nar/gkae358.

Stem Cell Reports, Volume *20*

Supplemental Information

Characterizing hypoxia-orchestrated post-stroke changes in oligodendrocyte precursor cells for optimized cell therapy

Yasuhiro Kuwata, Ken Yasuda, Kazuto Tsukita, Akihiro Kikuya, Naoki Takayama, Narufumi Yanagida, Kimitoshi Kimura, Ryosuke Takahashi, Riki Matsumoto, and Takakuni Maki

Supplementary Methods

Quality control of single cell RNA sequencing (scRNAseq) data

We conducted a stringent and uniform quality control process for our data according to the single-cell best practices (Heumos et al., 2023), following these steps:

1. Filtering empty droplets: We used the “filtered_bc_matrix” from the Cell Ranger output.
2. Low-quality cell filtering: Outliers were excluded based on the number of detected genes, UMI counts, and mitochondrial percentage, considering values outside the median ± 5 median absolute deviations (MAD).
3. Ambient RNA correction: We used the “raw_bc_matrix” from the Cell Ranger output and corrected for ambient RNA using the SoupX package (version 1.6.2) (Young and Behjati, 2020).
4. Preliminary doublet removal: We removed potential doublets using the scDblFinder package (version 1.14.0) (Germain et al., 2021).

After quality control, we performed count normalization using the “NormalizeData” function in the Seurat package (version 5.0.1), specifying the LogNormalize method with a scale factor of 10,000. We then selected 3,000 variable genes for integration using the “SelectIntegrationFeatures” function in Seurat (version 5.0.1) and integrated all datasets using Harmony (version 1.2.0) (Korsunsky et al., 2019). We applied Uniform Manifold Approximation and Projection (UMAP) dimensionality reduction using Harmony-corrected axes, removed remaining droplets, and annotated cell types based on specific cell markers. Additionally, based on the final annotation, we excluded any datasets containing fewer than 10 Oligodendrocyte Precursor Cells (OPCs). Finally, we conducted unsupervised clustering using the “FindClusters” function (resolution: 1) in the Seurat package (version 5.0.1).

Analyses of transient middle cerebral artery occlusion (tMCAO) atlas

We assessed the function of individual cells by scoring their expression of gene signatures associated with specific biological functions. Gene signatures were primarily retrieved from MSigDB using the msigdbr R package (version 7.5.1) (Castanza et al., 2023) and from the CollecTRI database, which provides comprehensive information on transcription factor regulons (Müller-Dott et al., 2023). To functionally characterize OPCs, we used OPC migration/myelination-associated gene sets (Hamanaka et al., 2023) and OPC-specific HIF-1α-target gene sets (Allan et al., 2021). We also reanalyzed microarray datasets from Olig2cre-DA-cat mice to extract the top 10 differentially expressed genes (DEGs) defining WNT-activated OPCs (Fancy et al., 2009, 2014; Tsai et al., 2016). For defining *ex vivo* severe-hypo-OPCs and mild-hypo-OPCs, we extracted the top 10 DEGs and genes with an adjusted P value < 0.05, respectively. Gene-set scores were calculated using the “AddModuleScore_UCell” function in the UCell R package (version 2.4.0) (Andreatta and Carmona, 2021). For heatmaps, median UCell scores were calculated, normalized, and visualized with color scaling to highlight differences between groups. Statistical analyses were performed using Wilcoxon rank-sum tests, with multiple testing corrections applied using the Benjamini-Hochberg method. For DEG identification, we used the “FindMarkers” function in the Seurat package (version 5.0.1), specifying test.use = “MAST” and min.pct = 0.1 (Finak et al., 2015). To infer cell-cell interactions, we applied the CellChat R package (version 1.6.1) according to its official tutorial (Jin et al., 2023). For trajectory inference, we first conducted a diffusion map using the “scanpy.tl.diffmap” function in Scanpy (version 1.9.6) in Python 3 (version 3.10.13) (Wolf et al.,

2018), followed by trajectory inference and diffusion map pseudotime calculation using the Slingshot R package (version 2.8.0) (Street et al., 2018).

RNA-seq library preparation and sequencing

cDNA libraries were prepared using the Agilent SureSelect Strand Specific RNA Prep Kit (Catalog no. G9691A) with 200 ng of total RNA, following the manufacturer's guidelines. Sequencing was performed on an Illumina MiSeq platform, generating 76 bp paired-end reads with multiplexing.

Processing of bulk RNAseq data

We also applied uniform and stringent data processing to all bulk RNA-seq datasets. Adapter sequences were removed, and reads were quality-trimmed using Trimmomatic (version 0.36). Post-trimming, read quality was assessed with FastQC (version 0.11.9). The primary assembly version of the Rattus norvegicus reference genome (mRatBN7.2) was obtained from the Ensembl database. Trimmed reads were then aligned to the reference genome using STAR aligner (version 2.7.9a), and gene expression levels were quantified using RSEM (version 1.3.3).

Analyses of bulk RNAseq data

For differential gene expression analysis, we used the edgeR package (version 3.42.4). Gene counts were normalized using the "calcNormFactors" function, and low-expression genes were filtered by retaining only those with a counts-per-million value above 10 in at least one sample. A linear model was fitted using the voom method from the limma package (version 3.56.2), and differential expression was tested using an empirical Bayes approach. For gene set enrichment

analysis (GSEA), gene signatures were extracted as detailed in the "Analyses of tMCAO Atlas" subsection and analyzed using the "GSEA" function from the clusterProfiler R package (version 4.8.3).

Other publicly available data analyzed in this study

In addition to the data used to create the tMCAO atlas, we analyzed the following scRNA-seq and bulk RNA-seq datasets:

• GSE114609: A bulk RNA-seq dataset of ex vivo severe-hypo-OPCs previously generated by our group (Kishida et al., 2019).

• GSE19403: A microarray dataset of *in vivo* OPCs from WNT-β-catenin constitutively activated mice (Fancy et al., 2009, 2014).

• GSE75330: An scRNA-seq dataset of *in vivo* OPCs from spinal cords (Marques et al., 2016).

• GSE173182: A bulk RNA-seq dataset of *ex vivo* OPCs treated with IFN-γ or dexamethasone (Meijer et al., 2022).

• Multi-organ endothelial cell atlas: An scRNA-seq dataset of endothelial cells from seven organs (Bondareva et al., 2022).

For these datasets, we downloaded the corresponding FASTQ files and processed them through the pipeline described earlier.

Supplementary References

Allan, K.C., Hu, L.R., Scavuzzo, M.A., Morton, A.R., Gevorgyan, A.S., Cohn, E.F., Clayton, B.L.L., Bederman, I.R., Hung, S., Bartels, C.F., et al. (2021). Non-canonical targets of HIF1a impair oligodendrocyte progenitor cell function. Cell Stem Cell 28, 257–272.e11.

Andreatta, M., and Carmona, S.J. (2021). UCell: robust and scalable single-cell gene signature scoring. Comput. Struct. Biotechnol. J. 19, 3796–3798.

Bondareva, O., Rodríguez-Aguilera, J.R., Oliveira, F., Liao, L., Rose, A., Gupta, A., Singh, K., Geier, F., Schuster, J., Boeckel, J.-N., et al. (2022). Single-cell profiling of vascular endothelial cells reveals progressive organ-specific vulnerabilities during obesity. Nat. Metab. 4, 1591–1610.

Castanza, A.S., Recla, J.M., Eby, D., Thorvaldsdóttir, H., Bult, C.J., and Mesirov, J.P. (2023). Extending support for mouse data in the Molecular Signatures Database (MSigDB). Nat. Methods 20, 1619–1620.

Fancy, S.P.J., Baranzini, S.E., Zhao, C., Yuk, D.-I., Irvine, K.-A., Kaing, S., Sanai, N., Franklin, R.J.M., and Rowitch, D.H. (2009). Dysregulation of the Wnt pathway inhibits timely myelination and remyelination in the mammalian CNS. Genes Dev. 23, 1571–1585.

Fancy, S.P.J., Harrington, E.P., Baranzini, S.E., Silbereis, J.C., Shiow, L.R., Yuen, T.J., Huang, E.J., Lomvardas, S., and Rowitch, D.H. (2014). Parallel states of pathological Wnt signaling in neonatal brain injury and colon cancer. Nat. Neurosci. 17, 506–512.

Finak, G., McDavid, A., Yajima, M., Deng, J., Gersuk, V., Shalek, A.K., Slichter, C.K., Miller, H.W., McElrath, M.J., Prlic, M., et al. (2015). MAST: a flexible statistical framework for assessing transcriptional changes and characterizing heterogeneity in single-cell RNA sequencing data. Genome Biol. 16, 278.

Germain, P.-L., Lun, A., Garcia Meixide, C., Macnair, W., and Robinson, M.D. (2021). Doublet identification in single-cell sequencing data using scDblFinder. F1000Res 10, 979.

Hamanaka, G., Hernández, I.C., Takase, H., Ishikawa, H., Benboujja, F., Kimura, S., Fukuda, N., Guo, S., Lok, J., Lo, E.H., et al. (2023). Myelination- and migration-associated genes are downregulated after phagocytosis in cultured oligodendrocyte precursor cells. J. Neurochem. 167, 571–581.

Heumos, L., Schaar, A.C., Lance, C., Litinetskaya, A., Drost, F., Zappia, L., Lücken, M.D., Strobl, D.C., Henao, J., Curion, F., et al. (2023). Best practices for single-cell analysis across modalities. Nat. Rev. Genet. 24, 550–572.

Jin, S., Plikus, M.V., and Nie, Q. (2023). CellChat for systematic analysis of cell-cell communication from single-cell and spatially resolved transcriptomics. bioRxiv. https://doi.org/10.1101/2023.11.05.565674

Kishida, N., Maki, T., Takagi, Y., Yasuda, K., Kinoshita, H., Ayaki, T., Noro, T., Kinoshita, Y., Ono, Y., Kataoka, H., et al. (2019). Role of perivascular oligodendrocyte precursor cells in angiogenesis after brain ischemia. J. Am. Heart Assoc. 8, e011824.

Korsunsky, I., Millard, N., Fan, J., Slowikowski, K., Zhang, F., Wei, K., Baglaenko, Y., Brenner, M., Loh, P.-R., and Raychaudhuri, S. (2019). Fast, sensitive and accurate integration of single-cell data with Harmony. Nat. Methods 16, 1289–1296.

Marques, S., Zeisel, A., Codeluppi, S., van Bruggen, D., Mendanha Falcão, A., Xiao, L., Li, H., Häring, M., Hochgerner, H., Romanov, R.A., et al. (2016). Oligodendrocyte heterogeneity in the mouse juvenile and adult central nervous system. Science 352, 1326–1329.

Meijer, M., Agirre, E., Kabbe, M., van Tuijn, C.A., Heskol, A., Zheng, C., Mendanha Falcão, A., Bartosovic, M., Kirby, L., Calini, D., et al. (2022). Epigenomic priming of immune

genes implicates oligodendroglia in multiple sclerosis susceptibility. Neuron 110, 1193–1210.e13.

Müller-Dott, S., Tsirvouli, E., Vazquez, M., Ramirez Flores, R.O., Badia-I-Mompel, P., Fallegger, R., Türei, D., Lægreid, A., and Saez-Rodriguez, J. (2023). Expanding the coverage of regulons from high-confidence prior knowledge for accurate estimation of transcription factor activities. Nucleic Acids Res. 51, 10934–10949.

Street, K., Risso, D., Fletcher, R.B., Das, D., Ngai, J., Yosef, N., Purdom, E., and Dudoit, S. (2018). Slingshot: cell lineage and pseudotime inference for single-cell transcriptomics. BMC Genomics 19, 477.

Tsai, H.-H., Niu, J., Munji, R., Davalos, D., Chang, J., Zhang, H., Tien, A.-C., Kuo, C.J., Chan, J.R., Daneman, R., et al. (2016). Oligodendrocyte precursors migrate along vasculature in the developing nervous system. Science 351, 379–384.

Wolf, F.A., Angerer, P., and Theis, F.J. (2018). SCANPY: large-scale single-cell gene expression data analysis. Genome Biol. 19, 15.

Young, M.D., and Behjati, S. (2020). SoupX removes ambient RNA contamination from droplet-based single-cell RNA sequencing data. Gigascience 9, giaa151.

Table S1. Single-cell RNA sequencing datasets considered in this paper.

BioProject, project ID	Sample ID	Mouse (strain, genotype, age, sex)	Characteristics	FACS	Ischemia duration	Included?
PRJNA597034	GSM4227433	C57BL/6, WT, 6 weeks, male	Contralateral	Not applied	1.0 hour	No[1]
PRJNA597034	GSM4227434	C57BL/6, WT, 6 weeks, male	post-tMCAO 4 hrs	Not applied	1.0 hour	No[2]
PRJNA597034	GSM4227435	C57BL/6, WT, 6 weeks, male	Contralateral	Not applied	1.0 hour	No[1]
PRJNA597034	GSM4227436	C57BL/6, WT, 6 weeks, male	post-tMCAO 1 days	Not applied	1.0 hour	Yes
PRJNA597034	GSM4227437	C57BL/6, WT, 6 weeks, male	Contralateral	Not applied	1.0 hour	No[1]
PRJNA597034	GSM4227438	C57BL/6, WT, 6 weeks, male	post-tMCAO 3 days	Not applied	1.0 hour	Yes
PRJNA597034	GSM4227439	C57BL/6, WT, 6 weeks, male	Contralateral	Not applied	1.0 hour	No[1]
PRJNA597034	GSM4227440	C57BL/6, WT, 6 weeks, male	post-tMCAO 7 days	Not applied	1.0 hour	Yes
PRJNA597034	GSM4227441	C57BL/6, WT, 6 weeks, male	Sham	Not applied	–	Yes
PRJNA597034	GSM4227442	C57BL/6, WT, 6 weeks, male	Sham	Not applied	–	Yes
PRJNA597034	GSM4227443	C57BL/6, Cd300a-fl/f, 6 weeks, male	Sham	Not applied	–	Yes
PRJNA646185	GSM4670418	C57BL/6, Cd300a-fl/f, NS, NS	Sham	CD45-positive	–	No[3]
PRJNA646185	GSM4670420	C57BL/6, Cd300a-fl/f, NS, NS	post-tMCAO 1 hr	CD45-positive	1.0 hour	No[3]
PRJNA646185	GSM4670422	C57BL/6, Cd300a-fl/f, NS, NS	post-tMCAO 3 hrs	CD45-positive	1.0 hour	No[3]
PRJNA705094	GSM5111158	C57BL/6, WT, 10 weeks, male	Sham	Not applied	–	Yes
PRJNA705094	GSM5111159	C57BL/6, WT, 10 weeks, male	post-tMCAO 14 days	Not applied	1.5 hour	Yes
PRJNA718585	GSM5220257	C57BL/6, WT, NS, NS	post-tMCAO 5 days	CD45-positive	1.0 hour	No[3]
PRJNA718585	GSM5220258	C57BL/6, WT, NS, NS	post-tMCAO 5 days	CD45-positive	1.0 hour	No[3]
PRJNA718585	GSM5220259	C57BL/6, WT, NS, NS	post-tMCAO 5 days	CD45-positive	1.0 hour	No[3]
PRJNA718585	GSM5220260	C57BL/6, WT, NS, NS	post-tMCAO 5 days	CD45-positive	1.0 hour	No[3]
PRJNA730565	GSM5319987	C57BL/6, WT, 6-8 weeks, NS	Sham	Not applied	–	Yes
PRJNA730565	GSM5319988	C57BL/6, WT, 6-8 weeks, NS	Sham	Not applied	–	Yes

PRJNA730565	GSM5319989	C57BL/6, WT, 6-8 weeks, NS	Sham	Not applied	–	Yes
PRJNA730565	GSM5319990	C57BL/6, WT, 6-8 weeks, NS	post-tMCAO 1 day	Not applied	1.0 hour	Yes
PRJNA730565	GSM5319991	C57BL/6, WT, 6-8 weeks, NS	post-tMCAO 1 day	Not applied	1.0 hour	Yes
PRJNA730565	GSM5319992	C57BL/6, WT, 6-8 weeks, NS	post-tMCAO 1 day	Not applied	1.0 hour	Yes
PRJNA783004	GSM5701739	C57BL/6, WT, NS, NS	Sham	CD45-positive	–	No[3]
PRJNA783004	GSM5701741	C57BL/6, WT, NS, NS	Sham	CD45-positive	–	No[3]
PRJNA783004	GSM5701742	C57BL/6, WT, NS, NS	post-tMCAO 1 day	CD45-positive	0.5–0.75 hours	No[3]
PRJNA783004	GSM5701746	C57BL/6, WT, NS, NS	post-tMCAO 3 day	CD45-positive	0.5–0.75 hours	No[3]
PRJNA811768	GSM5701743	C57BL/6, WT, NS, NS	post-tMCAO 1 day	Not applied	1.0 hour	No[3]
PRJNA811768	GSM5929214	C57BL/6, WT, NS, NS	post-tMCAO 1 day	Not applied	1.0 hour	No[3]
PRJNA811768	GSM5929217	C57BL/6, WT, NS, NS	Sham	Not applied	–	No[3]
PRJNA811768	GSM5929218	C57BL/6, WT, NS, NS	post-tMCAO 2 days	Not applied	1.0 hour	No[3]
PRJNA858806	GSM6339290	C57BL/6, WT, NS, NS	Contralateral	A2B5, O4, CD45-positive cells	1.0 hour	No[1]
PRJNA858806	GSM6339291	C57BL/6, WT, NS, NS	post-tMCAO 14 days	A2B5, O4, CD45-positive cells	1.0 hour	Yes
PRJNA946281	GSM7104632	C57BL/6, WT, 8 weeks, NS	post-tMCAO 1 day	Not applied	1.5 hour	Yes
PRJNA946281	GSM7104633	C57BL/6, WT, 8 weeks, NS	post-tMCAO 3 days	Not applied	1.5 hour	Yes
PRJNA946281	GSM7104634	C57BL/6, WT, 8 weeks, NS	post-tMCAO 7 days	Not applied	1.5 hour	Yes
PRJNA946281	GSM7104635	C57BL/6, WT, 8 weeks, NS	Sham	Not applied	–	Yes
PRJEB42257	SAMEA7747040	C57BL/6, WT, NS, male	Sham	CD31/45-negative	–	Yes
PRJEB42257	SAMEA7747041	C57BL/6, WT, NS, male	post-tMCAO 3 days	CD31/45-negative	NS	Yes

[1]Contralateral hemisphere, [2]No other datasets collected in this time point, [3]Not sufficient OPCs.
Abbreviations: FACS, Fluorescence-Activated Cell Sorting; WT, Wild Type; NS, Not Specified; tMCAO, transient Middle Cerebral Artery Occlusion.

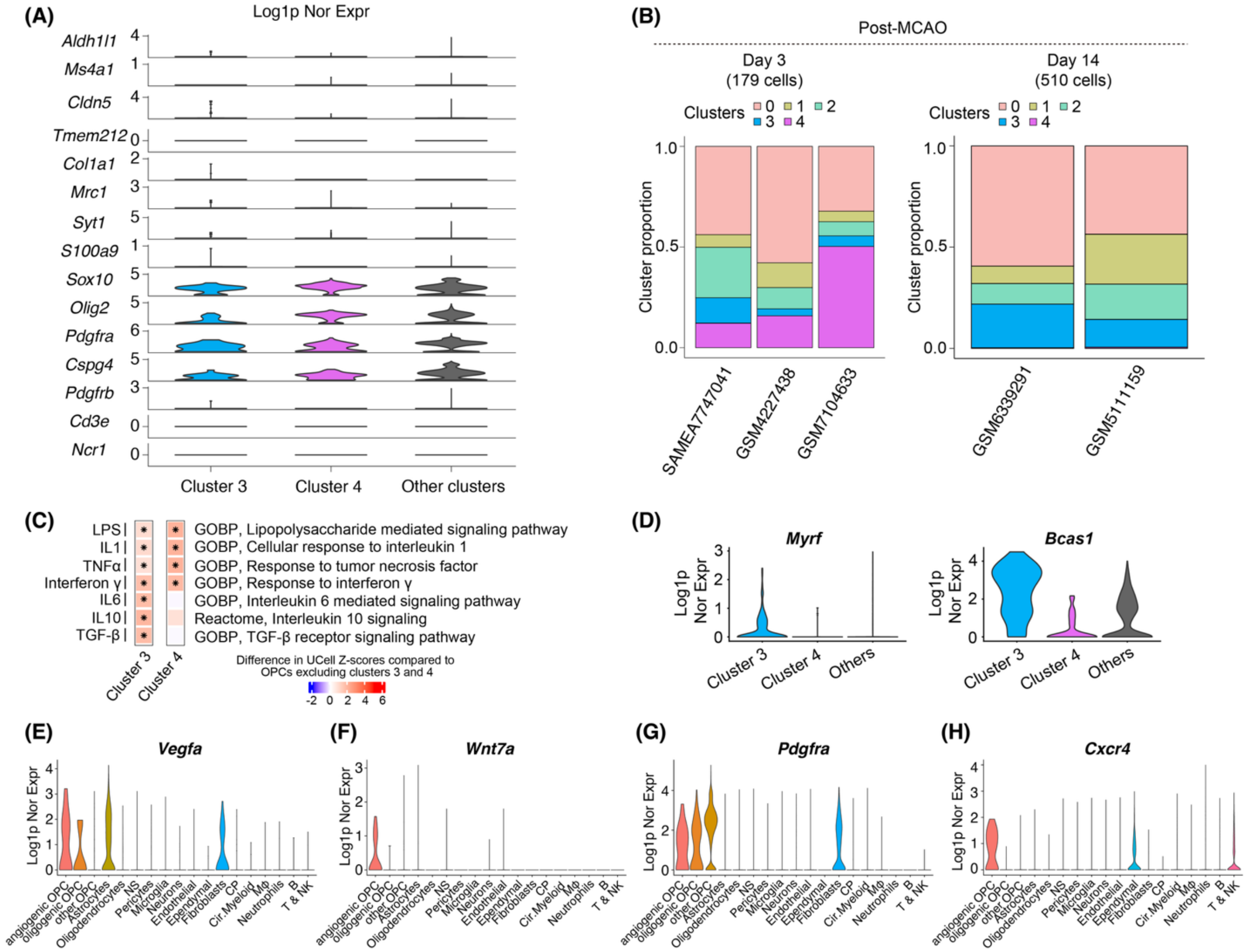

Fig. S1. Characterization of clusters 3 and 4 within oligodendrocyte precursor cells (OPCs). (A) OPC subclusters, including clusters 3 and 4, do not express genes specific to non-OPC cell types. (B) An increase in the proportion of clusters 3 and 4 is observed across all samples, with cluster 4 expanding at 3 days post-transient middle cerebral artery occlusion (tMCAO) and cluster 3 at 14 days post-tMCAO. Abbreviations: Log1p Nor Expr, Log1p-transformed normalized expression. (C) Both clusters are significantly influenced by various cytokines. Notably, cluster 4, corresponding to “angiogenic” OPCs, is more affected by cytokines involved in the acute inflammatory response, such as interleukin (IL)-1β and tumor necrosis factor (TNF)-α. In contrast, cluster 3, corresponding to “oligogenic” OPCs, is specifically influenced by IL-

10 and TGF-β. (D) *Myrf* and *Bcas1*, genes reported to be crucial for OPC maturation, are specifically expressed in cluster 3, with *Myrf* showing cluster-specific expression and Bcas1 being upregulated. (E, F) Cluster 4 of OPCs, corresponding to "angiogenic" OPCs, upregulates *Vegfa* (E) and *Wnt7a* (F), even compared to all other cell types. (G, H) *Pdgfra*, a marker gene for OPCs (G), and *Cxcr4*, a marker gene distinguishing "angiogenic" OPCs from other OPC types (H), distinctively mark "angiogenic" OPCs. Abbreviations: Log1p Nor Expr, Log1p-transformed normalized expression; GOBP, Gene Ontology Biological Process; Reactome, Reactome pathway database; NS, Neural Stem cells; CP, Choroid Plexus cells; Cir.Myeloid, Circulating Myeloid cells; Mφ, Macrophages; B, B cells; T & NK, T cells and NK cells.

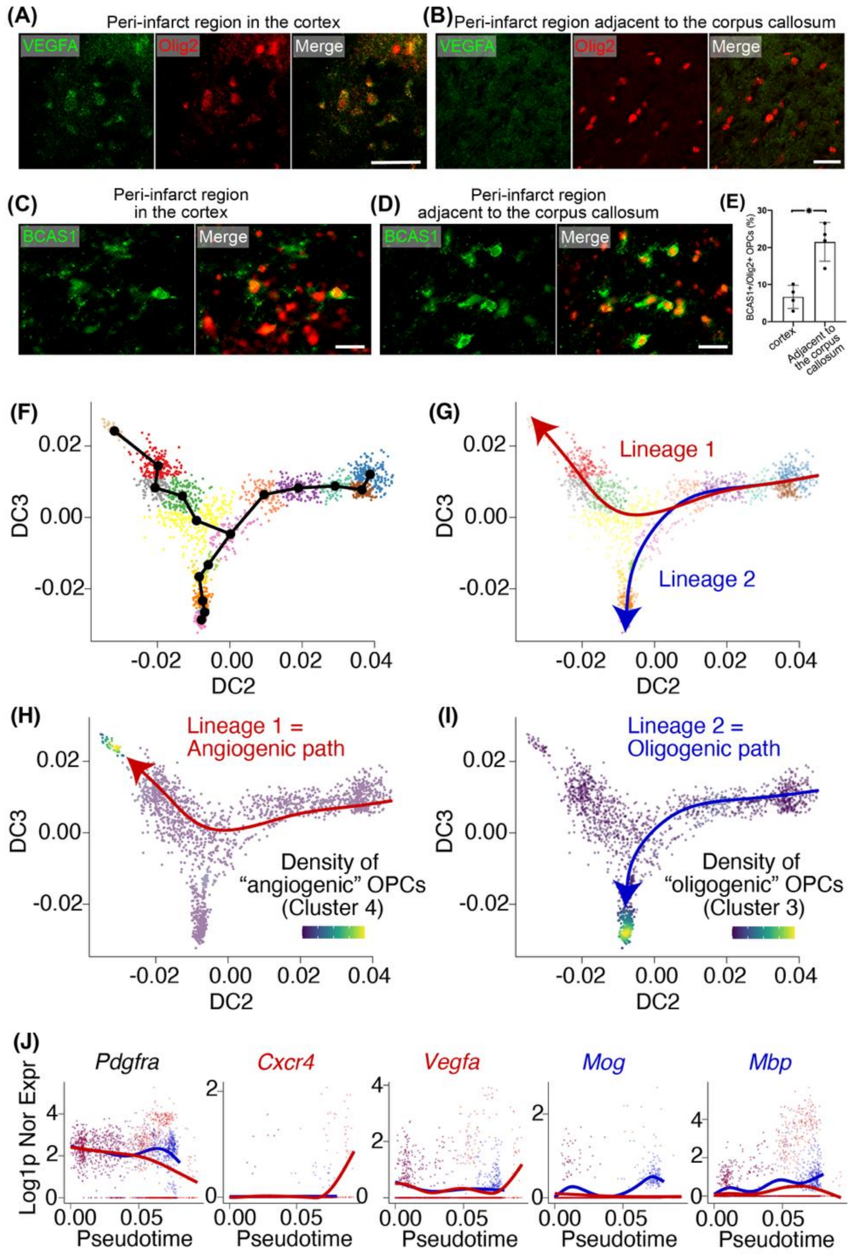

Fig. S2. Spatial distribution and trajectory analysis of angiogenic and oligogenic oligodendrocyte precursor cells (OPCs) following transient middle cerebral artery occlusion (tMCAO). (A, B) Representative immunofluorescence images showing VEGFA$^+$ OLIG2$^+$ "angiogenic" OPCs at day 3 post-tMCAO in the peri-infarct cortex (A) and the peri-infarct corpus callosum with its adjacent regions (B). (C, D) Representative immunofluorescence

images showing BCAS1$^+$ OLIG2$^+$ “oligogenic” OPCs at day 14 post-MCAO in the peri-infarct cortex (C) and the peri-infarct corpus callosum with its adjacent regions (D). (E) Quantification of the proportions of BCAS1$^+$ OLIG2$^+$ “oligogenic” OPCs (day 14) among total OLIG2$^+$ oligodendrocyte lineage cells in the peri-infarct cortex versus peri-infarct corpus callosum with its adjacent regions. N = 4. Scale bars = 50 µm. (F) Dimensional reduction using diffusion maps, followed by trajectory inference, identified a single branching point. (G) The “getCurves” function in the Slingshot R package revealed two distinct paths. (H, I) One path terminates in angiogenic OPCs, while the other terminates in oligogenic OPCs. (J) Gene expression changes along the diffusion map pseudotime show an increase in *Cxcr4* and *Vegfa* along the angiogenic path, whereas *Mog* and *Mbp* increase along the oligogenic path. Abbreviations: DC, Diffusion Component; Log1p Nor Expr, Log1p-transformed normalized expression.

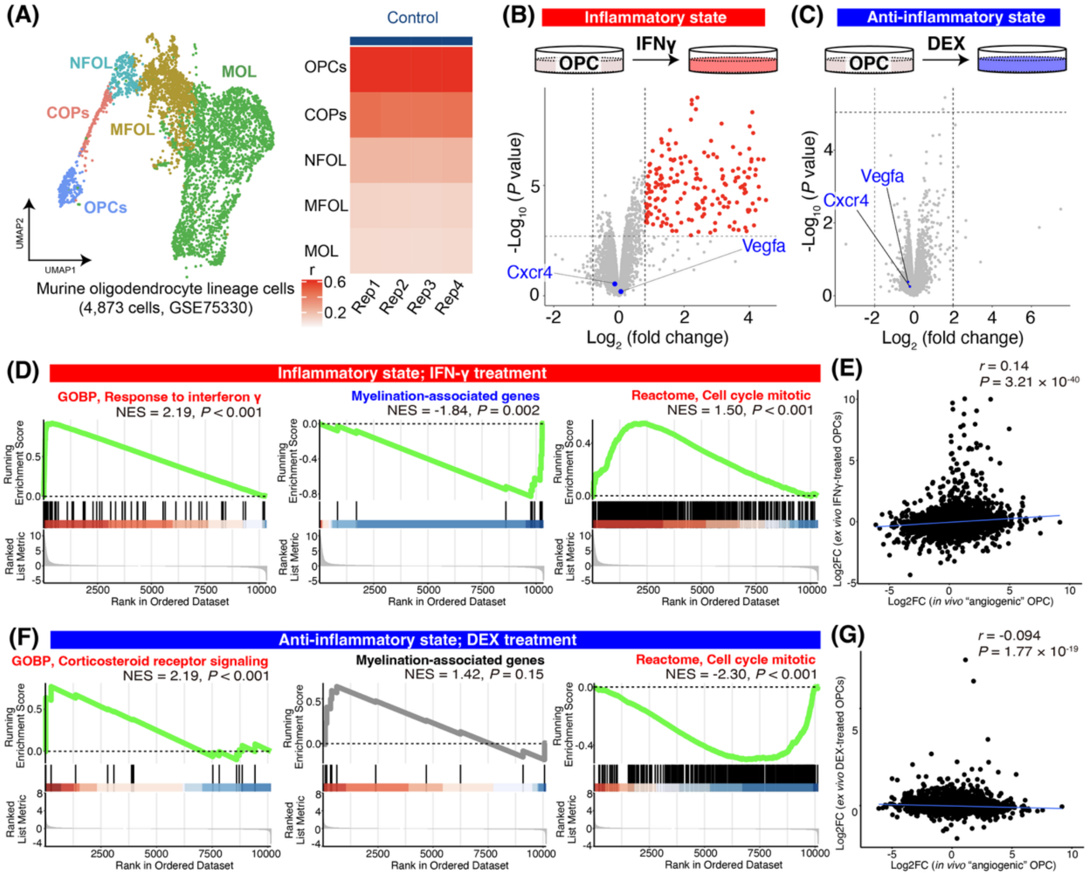

Fig. S3. Validation of *ex vivo* oligodendrocyte precursor cells (OPC) cultures and their transcriptomic responses to inflammatory and anti-inflammatory states. (A) Correlation analysis revealed that our *ex vivo* OPC cultures accurately capture the transcriptional profile of *in vivo* OPCs in the murine brain. (B, C) Inflammatory and anti-inflammatory states were induced using interferon-γ (IFN-γ) and dexamethasone (DEX), respectively. Differentially expressed gene (DEG) analysis revealed no significant transcriptomic changes in *Vegfa* and *Cxcr4*. (D) The inflammatory state led to the downregulation of myelination-associated genes and the upregulation of mitosis-associated genes. (E) Correlation analysis revealed weak but significant positive correlations of log2 fold changes (log2FC) between *in vivo* "angiogenic" OPCs and *ex vivo* IFN-γ-treated OPCs. (F) The anti-inflammatory state resulted in the downregulation of mitosis-associated genes. (G) Correlation analysis revealed weak but significant negative

correlations of log2FC between *in vivo* “angiogenic” OPCs and *ex vivo* DEX-treated OPCs. Abbreviations: GSE, Gene expression omnibus SEries; COPs, Committed Oligodendrocyte Precursors; NFOL, Newly Forming Oligodendrocytes; MFOL, Myelin-Forming Oligodendrocytes; MOL, Mature Oligodendrocytes; GOBP, Gene Ontology Biological Process; Reactome, Reactome pathway database; NES, Normalized Enrichment Score; *rho*, Spearman’s rank correlation coefficient.

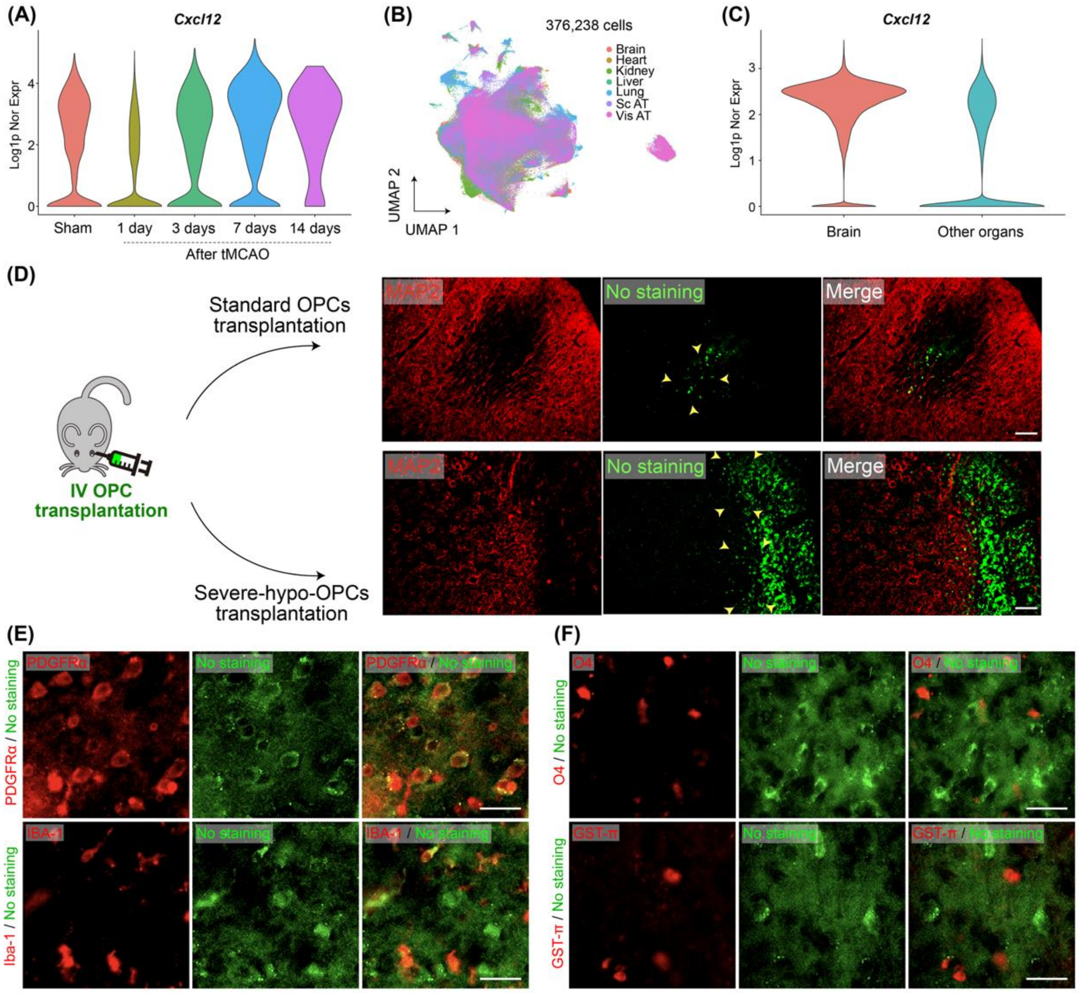

Fig. S4. Brain endothelial *Cxcl12* expression and behavior of transplanted oligodendrocyte precursor cells (OPCs) in the ischemic brain. (A) *Cxcl12* expression in endothelial cells was upregulated 3 days post-tMCAO. (B, C) Reanalysis of a publicly available cross-organ endothelial cell atlas (B) revealed that brain endothelial cells exhibit high *Cxcl12* expression (C). (D) Representative immunofluorescence images of EGFP-labeled standard OPCs and OPCs subjected to severe hypoxic preconditioning *ex vivo* (severe-hypo-OPCs), shown at day 14 after intravenous transplantation (3 days post-MCAO). GFP-positive transplanted OPCs were detected without additional anti-GFP staining ("No staining"). GFP^+ transplanted cells were observed in the ischemic border and peri-infarct penumbra (regions outlined by arrowheads). Please note that

the lesion shown in the upper panel represents a small remote infarct located slightly apart from the main ischemic core; this area was selected as representative because of its low background signal. Scale bar = 100 μm. (E) GFP-positive transplanted severe-hypo-OPCs were detected without additional anti-GFP staining (No staining), retained PDGFRα expression, and some were phagocytosed by IBA1-positive microglia. Scale bar = 30 μm. (F) GFP-positive transplanted severe-hypo-OPCs did not express oligodendrocyte maturation markers O4 and GST-π. Scale bar = 30 μm. Abbreviations: Log1p Nor Expr, Log1p-transformed normalized expression; Sc AT, Subcutaneous Adipose Tissue; Vis AT, Visceral Adipose Tissue.

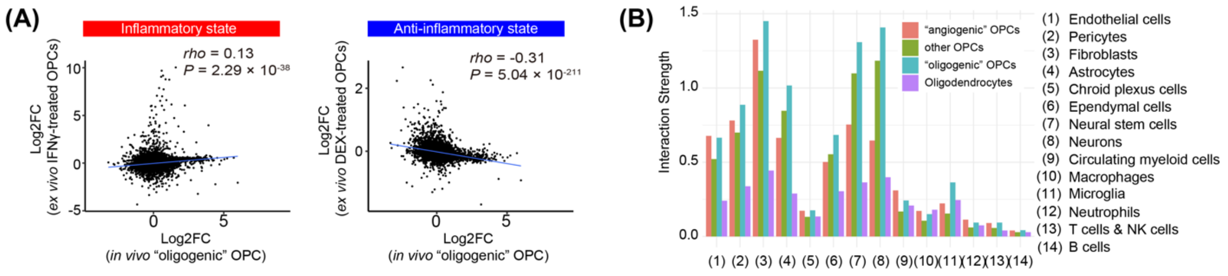

Fig. S5. The inflammatory state and cell-cell interactions may influence the transcriptomic characteristics of *in vivo* "oligogenic" oligodendrocyte precursor cells (OPCs). (A) Correlation analysis revealed significant positive correlations of $\log_2$ fold changes ($\log_2$FC) between in vivo "oligogenic" OPCs and ex vivo interferon-γ (IFN-γ)-treated OPCs, as well as notable negative correlations of log2FC between *in vivo* "oligogenic" OPCs and *ex vivo* dexamethasone (DEX)-treated OPCs. (B) Cell-cell interaction analysis revealed enhanced interactions between "oligogenic" OPCs and various cell types. Abbreviations: *rho*, Spearman's rank correlation coefficient.
